# Supplementary material for: Genome‐scale metabolic modeling reveals key features of a minimal gene set
Source: Mol Syst Biol. 2021 Jul 20;17(7):e10099. doi: 10.15252/msb.202010099 (PMC8290834; doi:10.15252/msb.202010099)
Supplement: Supplementary file 1 — Appendix [file MSB-17-e10099-s001.pdf]

Appendix

Genome-scale metabolic modeling reveals key features of a minimal gene set

Jean-Christophe Lachance[1], Dominick Matteau[1], Joëlle Brodeur[1], Colton J. Lloyd[2], Nathan Mih[2], Zachary A. King[2], Tom F. Knight[3], Adam M. Feist[2,5], Jonathan M. Monk[2], Bernhard O. Palsson[2,4,5,6], Pierre-Étienne Jacques[1] and Sébastien Rodrigue[1*]

Author affiliations

1. Département de Biologie, Université de Sherbrooke, Sherbrooke, Québec, Canada
2. Department of Bioengineering, University of California, San Diego, La Jolla, USA
3. Ginkgo Bioworks, Boston, Massachusetts, USA
4. Bioinformatics and Systems Biology Program, University of California, San Diego, La Jolla, USA
5. Department of Pediatrics, University of California, San Diego, La Jolla, CA, USA
6. Novo Nordisk Foundation Center for Biosustainability, Technical University of Denmark, Kemitorvet, Building 220, 2800 Kongens, Lyngby, Denmark

***Corresponding author. E-mail:** sebastien.rodrigue@usherbrooke.ca

This Appendix includes:

Appendix Supplementary Text

Appendix Figures S1-S10

Appendix Tables S1-S4

Appendix References

Table of contents

Appendix Supplementary Text 4
1. Identification of protein molecular functions in *M. florum* L1 4
1.1. Proteome comparison 4
1.2. Homology modeling 6
1.3. EC number identification 6
1.4. Final annotation score 7
2. Genome-scale metabolic network reconstruction 9
2.1. Nucleotides 10
2.2. Amino acids 16
2.3. Energy 17
2.4. Lipids 19
2.5. Glycans 23
2.6. Vitamins & Cofactors 25
3. *M. florum* growth medium 29
4. Conversion into a mathematical format 32
4.1. Biomass objective function 32
4.2. Sensitivity analysis 33
5. Validation of model phenotypic predictions 37
5.1. Carbohydrates utilization 37
5.2. Validation with proteomic and transcriptomic data 39
6. Model-driven prediction of a minimal genome 44
Appendix Figures 53
Appendix Tables 65
Appendix References 68

Appendix Supplementary Text

1. Identification of protein molecular functions in *M. florum* L1

Mesoplasma florum L1 is a near-minimal bacterium that was originally isolated from a lemon tree flower (McCoy *et al*, 1984). This microorganism belongs to the Mollicutes class, a group of small wall-less bacteria with genome sizes varying from ~560 kb in the case of *Mycoplasma genitalium* (Fraser *et al*, 1995) to more than a million (~1.5 Mbp) for *Acheloplasma laidlawii* (Lazarev *et al*, 2011) (Appendix Table S1). Due to their very small genomes, many Mollicutes represent interesting candidates for the study of the minimal components capable of sustaining cellular life (Morowitz, 1984). In that context, multiple studies have provided a general understanding of the metabolism of these near-minimal cells (Miles, 1992; Pollack & Williams, 1996). While this understanding is useful and has previously been leveraged for genome-scale metabolic reconstructions of different Mollicute species (Suthers *et al*, 2009; Wodke *et al*, 2013; Bautista *et al*, 2013), studies specifically interested in *M. florum* are rare. Hence, we set to extract a maximal amount of functional information from the *M. florum* genome by:

1. comparing the proteome of *M. florum* with predicted proteins encoded by Mollicutes for which genome-scale models (GEM) have been published,
2. extracting enzyme commission (EC) numbers,
3. and generating three-dimensional structures through homology modelling.

1.1. Proteome comparison

First, the *M. florum* proteome was compared with four Mollicutes species for which GEMs were previously generated (*Mycoplasma genitalium* (Suthers *et al*, 2009), *Mycoplasma pneumoniae* (Wodke *et al*, 2013), *Mycoplasma gallisepticum* (Bautista *et al*, 2013), and *Mycoplasma mycoides* JCVI-Syn3A (Breuer *et al*, 2019); see Table 1) using the PATRIC proteome comparison tool

(Wattam *et al*, 2017) (Figure 1A and B, Dataset EV1). Despite having the lowest total number of proteins (438), JCVI-syn3.0 had the highest number of orthologs (411) followed by *M. gallisepticum* (344), *M. pneumoniae* (326), and finally, *M. genitalium* (318) (Appendix Figure S1), which is consistent with the phylogenetic distances between *M. florum* and these organisms (Barré *et al*, 2004).

Gene names are useful to query public databases for putative functions but were initially scarcely assigned in *M. florum* with 96 occurrences out of 676 predicted proteins common to both RefSeq and PATRIC annotations (Dataset EV1). This comparison resulted in the association of 366 *M. florum* proteins to a gene name in at least one species (Figure 1E). Redundant gene name association across species provided higher confidence in these associations. Among the 281 gene names that were identified in more than one species, 156 were identical, 113 had two possible identifications (similar) and 12 had more than two different attributions. This approach allowed the identification of 72 new gene names in *M. florum*, considering that a total of 149 genes names are shared across all species surveyed and that 77 of those were already identified in *M. florum*. 15 of the remaining 19 gene names identified in *M. florum* were shared with at least one other species, while four gene names (*guaA*, *rpsT*, *rpmC*, *rpsF*) were specific to *M. florum*.

The proteome comparison approach allowed a link between proteins and model reaction identifiers, thereby generating an initial draft reconstruction of the *M. florum* metabolic network (Dataset EV1). The gene-reaction rule of each model allowed to link genes orthologous to *M. florum* to reactions in the models. The gene to reaction mapping obtained for each model was converted to BiGG identifiers (King *et al*, 2016) using MetaNetX (Moretti *et al*, 2016). This draft was used to initiate the metabolic reconstruction process. Identified reactions were added using the SimPheny software (Schilling *et al*) and later extracted to be used with the COBRApy toolbox (Ebrahim *et al*, 2013).

1.2. Homology modeling

Next, *M. florum* proteins were investigated from a structural standpoint. Given the unavailability of *M. florum* crystallized protein structures in the Protein Data Bank (PDB) (Berman *et al*, 2000), the Structural Systems Biology software (ssbio) (Mih *et al*, 2018) was used to map the 680 open reading frames predicted in the *M. florum* L1 genome annotation from RefSeq (NC_006055.1) against known domains in the PDB. Proteins containing successfully mapped domains were then selected for 3D homology modeling using the I-TASSER suite (Yang *et al*, 2015) (Figure 1C). Initially, 459 (67.5%) mapped to known domains in the PDB (Dataset EV2). After filtering, 386 domain-supported proteins were chosen for 3D structure prediction (Materials and Methods).

The quality of the generated 3D reconstruction is evaluated using the quality of the threading alignment and convergence of the assembly refinement performed by I-TASSER (Roy *et al*, 2010), summarized in a C-score and a TM-score (Appendix Figure S2). As recommended by the authors of the I-TASSER suite, we used a C-score cutoff of -1.5 and a TM-score cutoff of 0.5 to determine structures of higher quality. From the 386 proteins selected for homology modelling, 361 protein structures (95.6%) were deemed reliable (Figure 1E, Appendix Figure S2) and were further analyzed using COFACTOR, a software providing EC numbers, Gene Ontology (GO) terms, and binding site predictions (Zhang *et al*, 2017) (Figure 1C, Dataset EV2).

1.3. EC number identification

EC numbers provide valuable information on the biochemical reactions catalyzed by enzymes. EC numbers were obtained from both RefSeq and PATRIC genome annotations and compared to the predictions formulated by both COFACTOR and DETECT v2 (Figure 1D). Of the 393 proteins associated with at least one EC number, 207 were obtained with more than one method and 186 were identified with a single method (Dataset EV3). 112 of the 125 high-probability

identifications found by DETECT v2 were identical with at least one method. The remaining 13 identifications shared the first three EC digits with at least one other method. Of the 186 identifications found with a single method, 164 were specific to COFACTOR, 20 were specific to PATRIC and 2 were lower quality hits found only by DETECT v2. The consistency between EC number predictions was compared by matching the EC digits obtained with each method, showing that more than 87% have identical (113) or similar (67) EC digits (Figure 1E, Materials and Methods).

Our study is not the first to use *ad hoc* reconstruction of 3D structures for genome-wide identification of protein functions (Yang & Tsui, 2018; Yang *et al*, 2019; Antczak *et al*, 2019). Of all four approaches used, COFACTOR identified the most EC numbers (Figure 1D and Dataset EV3). Its predictions were nonetheless frequently different from standard, sequence-based methods. Albeit the potential for false positive identifications, these predictions were useful to formulate contextual hypotheses where the metabolic network suggested the need for a given function (see Figure 6). Faced with the great challenge of identifying several molecular functions required for synthetic biology, our study demonstrated the useful application of protein structures for the generation of testable hypotheses. With an increasing reliability of structure prediction algorithms (Billings *et al*, 2019; AlQuraishi, 2019; Senior *et al*, 2019), this type of approach is likely to gain interest.

1.4. Final annotation score

Our bioinformatic analysis allowed extracting extensive information from the genome and attributing a confidence score for each of those genes to function associations. While a similar approach using a combination of computational methods was previously used to predict unknown molecular functions in a genome (Ghatak *et al*, 2019), our rationale was that the cross-validations would increase confidence levels and establish a hierarchy in the current annotation (Figure 1E).

Indeed, most of the proteins for which no gene name was identified also did not map to known structural domains or functional EC number. Overall, between 283 and 315 proteins, ~45% of the total proteins had poor mappings to EC number and quality structures, respectively. Cross-validated top tier confidence proteins were scarcer with 156 identical gene names identified and 113 identical EC numbers identified through different methods which sums up to ~20% of the total proteins. The remaining ~35% of proteins had mixed identifications and cover the medium confidence range. To summarize the information contained in Figure 1E, a final annotation score was calculated (Materials and Methods and Figure 1F). These results demonstrate that the identification of molecular functions, even in small genomes, is far from complete. The high proportion of mitigated functions should stimulate the effort for experimental protein characterization (Glass *et al*, 2017).

2. Genome-scale metabolic network reconstruction

The reconstruction of the *M. florum* metabolic network was executed as described by Thiele and Palsson (Thiele & Palsson, 2010). To increase the reliability of the reconstruction, both GenBank and PATRIC (Wattam *et al*, 2017) genome annotations were used as reference annotations. The potential metabolic candidates were extracted based on EC numbers and product names. This information was used to query publicly available reaction databases (Kanehisa *et al*, 2017, 2016; Kanehisa & Goto, 2000; Placzek *et al*, 2017; Artimo *et al*, 2012). The identified reactions were added using the SimPheny framework to ensure charge balance and conformity with an existing functional nomenclature (Dataset EV1). Refinement of the initial reconstruction was made by curating each metabolic objective individually and studying literature for biochemical evidence in *M. florum*. An interactive map of the entire reconstructed *M. florum* metabolic network is provided in a json format as Computer Code EV1. The final *i*JI208 GEM is also provided in json format as Computer Code EV2.

The details of this manual reconstruction process are presented here and divided in six sections. Each section corresponds to a greater category named "Module". The utility of dividing the metabolism in such sections is to simplify future engineering tasks, a concept that was brought forth by Danchin and colleagues (Acevedo-Rocha *et al*, 2013; Danchin & Fang, 2016). The six modules presented here are: (1) Nucleotides, (2) Amino acids, (3) Energy, (4) Lipids, (5) Glycans, and (6) Vitamins & Cofactors (Figure 3). The details of every module composition together with the model in a spreadsheet format are available in Dataset EV4.

EC numbers and gene names identified through the computational identification of molecular functions were used to attribute reactions to the genes in the network. Along with our reasoning during the reconstruction of the model, the next sections identify genes for which the manual

curation raised questions or required comments. These areas of lesser knowledge would require further experimental biochemical characterization.

2.1. Nucleotides

The synthesis of nucleotides is fundamental to all life forms. Manual curation of the *M. florum* genome and identification of gene names and EC numbers revealed 44 genes associated with the Nucleotides module (Dataset EV4). This module is the largest by number of reactions but contains a lower number of genes (44) than the Energy module (57) (Figures 2 and 3). This module also holds the highest number of genes involved in multiple reactions (20). In particular, the pyruvate kinase (Mfl175) is involved in 10 reactions, the largest number for any gene in the model. Like in most Mollicutes, a dedicated nucleotide diphosphate kinase is absent in *M. florum* (Pollack *et al*, 2002), and the pyruvate kinase was hypothesized to generate nucleotide triphosphates for all nucleotides.

Ribose

Ribose, which serves as a backbone for nucleotides, has an associated ABC transporter encoded in the *M. florum* genome (Mfl666, Mfl667, Mfl668 and Mfl669). Intracellular ribose is then phosphorylated by a specific ribose kinase (Mfl642). It is noteworthy that, through the proteome comparison process, this protein matched with the *fruK* gene product of *M. genitalium*, a protein annotated as putative phosphofructokinase. In *M. florum*, both PATRIC and RefSeq annotations suggested a ribokinase activity with EC 2.7.1.15 (ribokinase or deoxy-ribokinase) identified by both PATRIC and COFACTOR. We corrected the gene name from *fruK* to *rbsK*, to be consistent with the *Escherichia coli* nomenclature (Dataset EV4).

Phosphoribosyltransferases

Phosphoribose can then be used in the pentose phosphate pathway (PPP) or in the nucleotide

synthesis. To be included into the synthesis of nucleotides, a nucleobase needs to be fixed to ribose. The enzyme that catalyzes this process for the various nucleobases is a phosphoribosyltransferase. Reviewing the annotation of *M. florum* highlighted a total four phosphoribosyltransferases (Mfl107, Mfl276, Mfl463, and Mfl588).

Mfl107 (*upp*) encodes a uracil phosphoribosyltransferase for which three methods assigned the EC number 2.4.2.9. The confidence is high that this enzyme is specific to uracil. The presence of these three phosphoribosyltransferases suggests that *M. florum* is capable of synthesizing every nucleic acid necessary for *de novo* synthesis of DNA and RNA from free nucleobases and ribose.

The EC number identification for Mfl276 (*apt*) was consistent in all four methods and converged on EC 2.4.2.7. The reaction catalyzed by this gene is adenine or adenosine phosphoribosyltransferase which fixes the nucleobase adenine to the first carbon of the phosphorylated ribose, generating a nucleotide.

The phosphoribosyltransferase Mfl463 (*hpt*) annotated in RefSeq is "hypoxanthine-guanine phosphoribosyltransferase". Three methods associated the same EC number to this gene (EC 2.4.2.8). The KEGG (Kanehisa *et al*, 2017) annotation states that the guanine phosphoribosyltransferase can use hypoxanthine as a substrate for the reaction. Two reactions were therefore associated with this gene in the model: GUAPRT and HXPRT.

The fourth enzyme of this class is the nicotinate phosphoribosyltransferase encoded by Mfl588 and is discussed further in the Vitamins & Cofactors module. The current RefSeq annotation identified the EC number 2.4.2.11 which is obsolete according to KEGG (Kanehisa *et al*, 2017). The replacement EC number (6.3.4.21) was correctly identified by both PATRIC and DETECT while COFACTOR also attributed the old EC number 2.4.2.11. Given the high confidence in the

EC number attributed to that gene we rename it *pncB* to be consistent with the *Salmonella typhimurium* annotation from which the function was fetched (Vinitsky & Grubmeyer, 1993).

DNA uptake

Previous studies have suggested that no transporter exists for nucleosides or nucleotides in Mollicutes (Pollack, 2002). Nevertheless, the manual curation of the genome allowed to identify two gene products that could satisfy the demand for individual nucleobases. Mfl413 and Mfl658 are both identified in RefSeq as Uracil/Xanthine permeases. The structure for a uracil permease (*uraA*) was previously generated (Lu *et al*, 2011) and the authors suggested a proton symport mechanism. The associated gene in *M. florum* and other Mollicutes is named *pyrP*. While the *E. coli* gene seemed specific to uracil, the current annotation for Mollicutes suggests the import of both a purine (Xanthine) and a pyrimidine (Uracil). Considering that those genes are the only two associated with nucleobases import in *M. florum* and the potential for promiscuity of reactions catalyzed by an organism whose genome has been reduced (Seelig, 2017), we initially included the import of all nucleobases for which a phosphoribosyltransferase reaction was annotated. Hereby, we identified adenine, guanine, xanthine/hypoxanthine and uracil as the first attempt at characterizing essential nucleobases for *M. florum*.

Another possible system worth mentioning is catalyzed by the DNA uptake proteins (Mfl027 and Mfl329). As suggested before (Bizarro & Schuck, 2007; Pollack *et al*, 1997), in the Mollicutes' natural environment, DNA uptake may occur through the direct import of larger fragments of DNA from nearby dying cells (Pollack, 2002). In a laboratory setting the long DNA fragments could come from undefined media components such as yeast extract (YE). These large fragments could then be digested using exonucleases. Mfl055 is a 5'-3' exonuclease that could be used for this process. While this mechanism remains hypothetical, the possibility that long DNA fragments

could be degraded by membrane associated nucleases and incorporated by a competency related protein should be kept in mind for further biochemical characterizations.

Finally, the whole network curation identified four putative essential components necessary for *M. florum* growth: adenine, guanine, thymidine and ribose. Although an entire ABC transport system is annotated for ribose (Mfl666, Mfl667, Mfl668), the exact nature and function for DNA uptake would require further characterization in a completely defined medium.

Phosphorylation of nucleotides

Monophosphate-nucleotides formed by the combination of the phosphorylated ribose backbone and the imported nucleobases need to be phosphorylated twice before they can be incorporated into macromolecules (DNA and RNA). The first phosphorylation step leads to the formation of diphospho-nucleotides. Adenylate kinase (Mfl144, *adk*), guanylate kinase (Mfl195, *gmk*), cytidylate kinase (Mfl198, *cmk*), uridine kinase (Mfl306, *udk*), and thymidylate kinase (Mfl676, *tmk*) activities are annotated in *M. florum* with consistent EC numbers found across three different methods for each of them (Datasets EV3 and EV4).

In *M. florum,* the phosphorylation of deoxy-nucleotides was not specifically reported by the RefSeq annotation. Nevertheless, by consulting the PATRIC annotation, deoxyadenosine kinase (EC 2.7.1.76) and deoxyguanosine kinase (EC 2.7.1.113) were attributed to Mfl547. The EC 2.7.1.113 was also reported by COFACTOR. Our approach also identified a specificity to deoxythymidine. In fact, a dTMP kinase activity (EC 2.7.4.9) was attributed to Mfl676 by PATRIC, DETECT and COFACTOR (Datasets EV3 and EV4).

In Mollicutes, the lack of annotation for a Nucleotide Diphosphate Kinase (NDPK) is common (Bizarro & Schuck, 2007; Pollack *et al*, 1997). It has been hypothesized that the relaxation of the

catalytic site of the glycolytic enzyme pyruvate kinase (Mfl175, *pyk*) would allow it to phosphorylate other nucleotides than ADP (Pollack *et al*, 2002). It has been reported that the Mollicute's *pyk* conserves 5 to 21% of its activity when using other substrates than ADP (Pollack *et al*, 2002). For modeling purposes, reactions PYK2 to PYK10 (eight reactions) were added to ensure that all nucleotide-diphosphate could be converted into nucleotide-triphosphate, building blocks of DNA and RNA.

Ribonucleoside-diphosphate reductase

The conversion between deoxy- and ribonucleotides is ensured by ribonucleosides-diphosphate reductases. *M. florum* encodes a thioredoxin (Mfl178, *trx*) and a thioredoxin reductase (Mfl064, *ntr)*. This system plays an important role in oxidoreductive balance and is present in Mollicutes (Ben-Menachem *et al*, 1997; Pollack *et al*, 1997). The conversion of all four nucleosides di-phosphate into nucleotides di-phosphate is likely to be catalyzed by the putative trimer complex formed by Mfl528 (*nrdA* or *nrdE*), Mfl529 (*nrdI*) and Mfl530 (*nrdF*) in a promiscuous manner.

Synthases

A GMP synthase (Mfl342, *guaA*) activity was identified through the four EC number identification methods used (EC 6.3.5.2). This enzyme enables the conversion of L-glutamate to L-glutamine, consuming one Xanthosine 5'-phosphate and producing one GMP. The fact that this enzyme is kept in *M. florum* may indicate either the requirement for an easy conversion between amino acids in case of starvation or the need to utilize non-conventional nucleotides like XMP.

A thymidylate synthase (Mfl419, *thyA*) activity was identified through the four EC number identification methods used (EC 2.1.1.45). This enzyme converts dUMP into dTMP using folate as a cofactor (5,10-Methylenetetrahydrofolate to 7,8-Dihydrofolate). This mechanism is likely conserved to ensure that accidental deoxidation of UMP into dUMP can be re-utilized.

A cytidine triphosphate synthetase (Mfl648, *pyrG*) activity (EC 6.3.4.2) was also identified through the four EC number identification methods used, namely RefSeq, PATRIC, DETECT v2, and COFACTOR (Datasets EV3 and EV4). This enzyme enables the production of CTP from UTP, converting glutamine into glutamate in the process. This process is likely conserved to ensure the availability of cytosine-based nucleotides and nucleosides. While the DNA uptake remains unclear given the current annotation of transporters, no specific transporter for cytosine was found.

Other nucleobases

Both Mfl074 and Mlf075 are annotated as adenylosuccinate lyases (EC 6.3.4.4 and EC 4.3.2.2, respectively). The reactions ADSS and ADSL1r convert aspartate to fumarate. In the metabolic network, fumarate is a dead-end metabolite. Further biochemical characterizations could link fumarate to other reactions in the network, hereby explaining the conservation of these enzymes.

An adenosine deaminase (Mfl215, *hit1*) matches both EC numbers 3.5.4.2 and 3.5.4.4. These are both responsible for the deamination of adenosine, producing either inosine or hypoxanthine and releasing ammonium. Both products are dead ends in the current metabolic network. One possible explanation for this reaction would be that un-orthodox nucleobases accumulating in the cell through various processes could be converted back to adenosine if this reaction was reversible.

A nucleotidyl hydrolase/transferase (Mfl245, *hit1*) was associated with two EC numbers 3.6.1.17 using PATRIC or 3.-.-.- with COFACTOR. The confidence in this annotation is weaker given that PATRIC identifies it as a Bis(5'-nucleosyl)-tetraphosphatase (asymmetrical). The EC number provided by COFACTOR gives only the first digit, which is not precise. Further experiments could

reveal the true activity of this enzyme.

2.2. Amino acids

De novo synthesis of amino acids is generally absent from Mollicutes species (Pollack, 2002), a feature that was also observed during the manual curation of *the M. florum* genome. Hence, salvage of free amino acids or oligopeptides appears as the only viable solution for *M. florum* to sustain protein production and growth. The possibility that both free amino acids and oligopeptides be imported in Mollicutes was previously discussed (Miles, 1992; Pollack, 2002; Yus *et al*, 2009). The Amino acids module is composed of two main transporter systems (single amino acid and peptides) that were suggested to import small peptides directly. These peptides are digested within the cell and the resulting amino acids are used to express proteins, a process that avoids the need for any energy expensive synthesis pathways. The apparent low number of transporters compared to the number of substrates (20 for all amino acids) has been suggested as biochemically possible (Hosie & Poole, 2001) and suiting the genome reduction history of Mollicutes (Pollack, 2002). Eight different gene products are annotated, revealing what could compose three different systems for amino acid and oligopeptides transport (Dataset EV4).

In *M. pneumoniae,* no amino acid can be synthesized *de novo,* and the defined growth medium previously developed by Yus and colleagues provides all amino acids (Yus *et al*, 2009). The decision was made to include exchange reactions for each amino acid into the *M. florum* model. For the import of oligopeptides, we referred to the strategy proposed for the *M. genitalium* model (*i*PS189) (Suthers *et al*, 2009). In *i*PS189, 15 dipeptide import reactions simulate the import of oligopeptides through oligopeptide ABC transporter and 14 reactions simulate the cleavage by a protease of these dipeptides into free amino acids that can be incorporated into proteins. These 29 reactions were imported from *i*PS189 and the gene-reaction rule was changed so that the Mfl094 to Mfl098 are associated with each of the import reactions. Eight proteases/dipeptidases

are annotated in *M. florum* based on GenBank (Dataset EV4).

Despite further evidence, all proteases but two were linked to these dipeptide cleavage reactions. The two proteases not taking part in cleavage of imported oligopeptides are the cell-division associated RasP/YluC (Mfl287), potentially involved in cell division, and the DNA-binding Lon protease (Mfl404) which could be linked to the heat-shock response.

The free amino acid import was hypothesized to be mediated via either Mfl605 or the complex formed by Mfl183 and Mfl184. Since Mfl183 and Mfl184 are annotated as hypothetical proteins in PATRIC, no further constraint was added through the addition of an ABC transport system requiring ATP. Instead, all free amino acid import reactions were considered to be proton symport.

2.3. Energy

The Energy module contains reactions associated with the production of ATP, alternate carbon metabolism reactions, oxidoreduction balance, pyruvate metabolism and an ATP pump. As in most Mollicutes (Miles, 1992), the tricarboxylic acid (TCA) cycle is absent from *M. florum* and glycolysis is the only ATP generating pathway, with lactate and acetate being the two possible fermentation by-products.

Phosphotransferase system (PTS)

It was previously reported that Mollicutes have the capacity to import and convert monosaccharides, and phosphorylate them upon entry (Pollack *et al*, 1983). This statement is consistent with the current genome annotation, which suggests that carbon sources can be incorporated via phosphotransferase systems (PTS) (see Figure 6B). Specificity was found for sucrose (Mfl516, Mfl527), glucose (Mfl187, Mfl214), fructose (Mfl181), trehalose (Mfl426, Mfl431, Mfl500) with the phosphotransferase (Mfl519) and phosphohistidine containing protein (Mfl565)

(Datasets EV3 and EV4). Two gene clusters ensure the transport of glycerol-3-phosphate (Mfl023, Mfl024, Mfl025, Mfl026) and ribose (Mfl666, Mfl667, Mfl668, Mfl669) through ABC transport.

Glycolysis

Although carbon sources utilized in glycolysis are predicted to be phosphorylated through PTS-associated transport, *M. florum*'s genome entails two sugar kinases. Glucose kinase (Mfl497) probably phosphorylates the remaining phosphate-free glucose molecule after trehalose is cleaved by trehalose-6-phopshate hydrolase. On the other hand, fructose kinase (Mfl514) most likely phosphorylates fructose after the sucrose molecule is cleaved by sucrose-6-phosphate hydrolase (Mfl515 or Mfl526). Aside from this initial phosphorylation step, *M. florum*'s glycolysis differs from some previously modelled Mollicutes at the glyceraldehyde-3-phosphate dehydrogenase step. In *M. florum* this enzyme has two versions. Mfl578 is annotated as the standard NAD dependent dehydrogenase converting glyceraldehyde-3-phosphate (g3p) into 3-phospho-glycerol phosphate (13dpg), a reducing reaction that produces NADH. The alternative reaction is catalyzed by gene Mfl259 (both PATRIC and GenBank annotations agree for NADP specificity) and converts g3p into 3-phospho-glycerate (3pg), bypassing phosphoglycerate kinase (Mfl577) reaction. This reaction utilizes NADP and generates NADPH.

ATPase pump

As for other Mollicutes, *M. florum* possesses an ATPase pump. Contrary to previous observations that the ATPase of Mollicutes is composed of seven genes (Béven *et al*, 2012), in *M. florum*, a cluster of eight genes (Mfl109 to Mfl116, inclusively) is proposed to form this complex. Unlike other bacteria where the F_1F_0 ATPase is used to generate energy from a proton gradient, in Mollicutes the ATPase is believed to be used by the cell to maintain an electro-chemical gradient at the cost of ATP. As previously reported the ATPase pump is also essential in *M. florum* with all

eight genes identified as essential and six of the eight genes untouched by any transposon (Dataset EV6). The first and the last gene in the genomic sequence were hit by a transposon only in the terminal part of the gene (the last 20%) which could still allow for the complex to form.

Secretion products

In *M. florum*, the enzyme lactate dehydrogenase (LDH: Mfl596) and the pyruvate dehydrogenase complex (PDH: Mfl039, Mfl040, Mfl041, and Mfl042) are annotated and would allow two outcomes for pyruvate. The first path through lactate leads to the production of NAD+ and lactate. NAD+ is used in glycolysis again whereas lactate needs to disappear from the system. No transporter was annotated for lactate, hence the orphan reaction L-LACt and the sink SK_L_LAC were added to the network creating an escape route for lactate. The PDH path leads to the formation of acetate for which no transporter was annotated either. Again, two orphan reactions were added to eliminate acetate from the system, a transport (ACtr) and a sink (SK_AC).

2.4. Lipids

The Lipids module contains the necessary machinery to synthesize the single *M. florum* cell membrane. Whole fatty acids are imported through two lipid transport proteins (Mfl590 and Mfl591). These fatty acids are then fixed to a glycerol backbone in a process dependent on the acyl-carrier protein (ACP, Mfl593). In the model, this generic glycerolipid is used to form the different lipid species previously detected in *M. florum* (Matteau *et al*, 2020).

Identification of lipid synthesis genes

The lipid synthesis network in *M. florum* was reconstructed using the available annotations (Datasets EV3 and EV4) and previously published experimental lipidomic data (Matteau *et al*, 2020). Most Mollicutes do not possess the ability to generate long chain fatty acid, an energy extensive process (Pollack *et al*, 1997). Lipid metabolism and requirements in Mollicutes is hard

to assess (Yus *et al*, 2009). Despite their genetic simplicity Mollicutes have conserved a rather high level of lipid complexity (Pollack *et al*, 1997). Although some studies have shown that *A. laidlawii* can execute *de novo* synthesis of fatty acids, the majority of less complex Mollicutes cannot execute this task since they appear to lack the necessary machinery, and also because the metabolic cost of fatty acid elongation (32 mole ATP/fatty acid) could be too high for these scavengers (Heath *et al*, 2002).

Experimental identification of lipid species

The previous characterization of the *M. florum* membrane composition by lipidomic (Matteau *et al*, 2020) was used as a guide for the identification of potential end goals of metabolic pathways. Nevertheless, these results were generated in the rich ATCC 1161 medium that contains undefined lipid species. The possibility that these lipids are residual from the undefined growth medium cannot be ruled out, even considering the efforts that were made to perform adequate washes of the cells before the experiment and the algorithmic noise reduction applied on these results. The lipidomic results were therefore evaluated when reconstructing the metabolic network and the reactions necessary for the production of these lipid species were added to the model (adding orphan or promiscuous reactions when necessary).

General mechanism

The mechanism for the production of lipid classes from Matteau and colleagues (Matteau *et al*, 2020) was assumed to be dependent on the Acyl-Carrier Protein (ACP, Mfl593). This highly conserved protein (Byers & Gong, 2007) can fix free fatty acids (FFA). The FFA transport system is potentially executed by Mfl590 and Mfl591, both annotated as “fatty acid binding/lipid transport protein” (GenBank) or a DegV family protein (PATRIC) in Pfam. The decision was made to use a single FFA (Octadecanoate (n-C18:0)) to serve as the fatty acid chain for all lipid classes in the model. The elongation of fatty acids is generally absent in Mollicutes (Pollack *et al*, 1997) and no

gene was identified that could catalyze this process. Since no elongation was modelled, the length of the fatty acid does not add a constraint on the system. If *M.florum* is presented with many different FFA in complex growth media, these FFA may be imported in the cell and next included in the cytoplasmic membrane. Upon activation of the ACP (Mfl384), the putative mechanism would involve the fixation of the FFA by an acyltransferase (Mfl607) yielding a FFA bound ACP. Fixation of the fatty acid chain to the glycerol backbone requires the production of a phosphorylated FFA (Mfl230) that can be fixed to the glycerol backbone (Mfl337). This FFA bound glycerol is converted into phosphatidic acid upon fixation of another fatty acid (Mfl382).

Phosphatidic acid derivatives

Previous experimental results suggested that *M. florum's* lipidic composition entails phosphatidylcholine, phosphatidylinositol-3-phosphate, phosphatidic acid and phosphatidylserine (PC, PIP3, PA and PS, respectively) (Matteau *et al*, 2020). The proposed general mechanism for assembly of lipids in *M. florum* would result in the formation of PA. The formation of the three other species (PC, PIP3 and PS) would result from the addition of the specific head on PA. Choline kinase was annotated in RefSeq but not in PATRIC. Three orphan reactions were added to fulfill the gap in choline production by the model (CHOLt, CHLPCTD, DAGCPT_mf). These reactions sequentially catalyze the fixation of choline-phosphate on CDP and further the fixation of choline from CDP-choline onto diacylglycerol (12dgr180_c). A similar modelling decision was taken for the formation of PIP3 where no transporter exists for inositol and the fixation of inositol on CDP requires the addition of an orphan reaction (CDIPT). PS also does not have an annotated gene for its synthesis from PA. While it is possible that the annotated Phosphatidylglycerol synthase (Mfl663) catalyzes this reaction, no evidence can confirm it. An orphan reaction (PSSA_mf) was therefore added for the synthesis of PS for *M. florum*.

Di-acylglycerol (DAG)

Diacylglycerol can be formed from PA. The current annotation does not contain any phosphatidate phosphatase that would be required for the synthesis of this metabolite. An orphan reaction (PAPA180) was added to satisfy this need.

Cardiolipin and phosphatidylglycerol

Cardiolipin is a component of cell membrane in all three domains of life (Schlame, 2008) and a ubiquitous component of the core biomass of prokaryotes as revealed by Xavier and colleagues (Xavier *et al*, 2017). While cardiolipin was not specifically identified in previous *M. florum* lipidomics experiments, Mfl626 is annotated as a cardiolipin synthase in both RefSeq and PATRIC. Phosphatidylglycerol (PG) was detected in lipidomics data and may be produced by *M. florum*. The presence of PG could be associated with cardiolipin due to its structure (also known as di-phosphoglycerol). The entire pathway for the synthesis of cardiolipin is annotated in *M. florum* so the reactions were added to the model.

Sphingomyelin

Sphingomyelin was ranked first in relative lipid abundance in previously generated *M. florum* lipidomic data (Matteau *et al*, 2020). Nevertheless, bacteria do not possess the capacity to produce sphingomyelin, an essential component of nerve tissue in mammalian cells (Oshida *et al*, 2003). Therefore, if this compound is present in the *M. florum* membrane it would be the result of a direct salvage from the environment. It has been reported that Mollicutes possess lipid salvage capability (Salman & Rottem, 1995; Saito *et al*, 1978). Sphingomyelin has also been shown to favor growth in a defined medium for some *Spiroplasma* species (Hackett *et al*, 1987). Despite these observations, no gene could be attributed to the import of sphingomyelin by *M. florum*. We hypothesized that the favored growth in presence of sphingomyelin was due to the increased lipid solubility which would facilitate the import of FFA from the medium. Given its high abundance in the published lipidomic dataset, sphingomyelin was added to the model and to the

BOF. Characterizing the cell membrane again, in a completely defined medium, would help determine the role and importance of sphingomyelin in *M. florum*.

2.5. Glycans

A similar data-driven approach was used for the reconstruction of the Glycans module, which contains the reactions responsible for the synthesis of the extracellular polysaccharide layer previously described for *M. florum* (Matteau *et al*, 2020). For modelling purposes, the synthesis of the capsular polysaccharides (CPS) was assumed to include the conversion of sugars (glucose, galactose, mannose and rhamnose) in a sugar-1-phosphate form and their fixation onto a nucleotide backbone. The only predicted glycosyltransferase (Mfl568) in *M. florum* was assumed to assemble the CPS directly on a diacylglycerol on the intracellular side of the membrane. The CPS is next transferred on the extracellular milieu by the flippase (Mfl562).

Many Mollicutes species have been reported to produce a thick layer of polysaccharides composed of neutral sugars, either attached to the membrane as CPS or secreted as extracellular polysaccharides (EPS) (Browning & Citti, 2014; Neyrolles *et al*, 1998; Bertin *et al*, 2013). For instance, Neyrolles and colleagues identified the thickness of the *Mycoplasma penetrans* CPS at 11-13 nm. In *M. florum*, transmission electron microscopy (TEM) showed a membrane thickness between 8 and 18 nm that would exceed the expected width for a single lipid bilayer (~4 nm) (Matteau *et al*, 2020). The composition of *M. penetrans* CPS assessed by gas liquid-chromatography revealed that it is composed of four different sugars: mannose, glucose, N-acetylglucosamine and N-acetylgalactosamine (Neyrolles *et al*, 1998). Like *M. penetrans*, GC-MS analysis of *M. florum* carbohydrates revealed four different monosaccharides (Matteau *et al*, 2020). Glucose and mannose were also found in *M. florum*, but the amino-sugars N-acetylglucosamine and N-acetylgalactosamine were not found. Instead, the two remaining components were identified as rhamnose and galactose.

The genome annotation of *M. florum* contains both a glucosyltransferase (Mfl568) and a *Wzx* flippase (Mfl562). In *E. coli* and *Salmonella* strains the pathway for O-antigen synthesis using Wzx/Wzy proteins can be summarized in a sequence of five events (Hong & Reeves, 2014):

1. The sugars are imported and phosphorylated in the process, usually via a PTS.
2. The phosphate group is transferred onto the first carbon, hereby labelling the sugar for polysaccharide synthesis.
3. The individual sugars are fixed to a triphosphate nucleotide via a nucleotidyltransferase.
4. The sugars are polymerized into a chain by a glycosyltransferase, using the energy contained in the phosphate bond with the nucleotide diphosphate.
5. The polymerized glycan is flipped on the extracellular side of the membrane by a flippase.

We suggested that a glucose transporter, either Mfl217 or Mfl187, could be promiscuous and allow the entry of sugar molecules composing the CPS. Interestingly, the *M. florum* GC-MS analysis also revealed the presence of rhamnose. While this sugar is similar to the other two, it lacks a hydroxyl group which is necessary for its phosphorylation upon entry. Therefore, the import of rhamnose was not associated with a gene and is included in the functions in search for a gene (Dataset EV4).

Sugars imported through the PTS should be phosphorylated on carbon 6. In order to be included in a polysaccharide, the phosphate group should be transferred on the first carbon. Mfl120 is annotated in RefSeq as a phosphomannomutase while being a D-Ribose 1,5-phosphomutase in PATRIC. Also, our re-annotation process allowed identifying three different EC numbers for this protein (Datasets EV3 and EV4). Together, these observations suggest that this enzyme is promiscuous (see Figure 6B). The conversion of hexose-6-phosphate to hexose-1-phosphate was therefore assigned to this gene for all sugars. Hexose-1-phosphate sugars are fixed to a

nucleotide-triphosphate via a nucleotidyl transferase/hydrolase. We suggest that Mfl245 occupies that function for all sugars.

Aside from the TEM images, one of the strongest evidences for the presence of CPS in *M. florum* is the annotation of both a glycosyltransferase (Mfl568) and a nearby O-antigen flipase/transporter (Mfl562). The addition of the first sugar to the diacylglycerol backbone and further elongation may be conducted by the only glycosyltransferase (Mfl568). Additional protein characterization may reveal the importance of other yet unannotated proteins in this process. Once the chain reaches a certain length, the instability may trigger the end of the elongation, preventing the attachment of the glycosyltransferase. The fully elongated chain may then be flipped on the other side of the membrane by the flippase (Mfl562). A polymer length of 13 nm may allow chains of seven or eight sugars. The proportions of each sugar identified in *M. penetrans* were 1:6:1:2 (mannose, glucose, N-acetylglucosamine, N-acetylgalactosamine). Here mannose, glucose, rhamnose, and galactose were identified through GC experiments in *M. florum* at a ratio of 1:4:4:11, respectively, and the CPS was incorporated in the model with this stoichiometry.

2.6. Vitamins & Cofactors

As for lipids and glycans, the synthesis of vitamins and cofactors in *M. florum* is very minimal. We describe here the pathways leading to the import and utilization of coenzymes that were identified in the annotation and used in the reconstruction process (Datasets EV3 and EV4).

Nicotinamide adenine dinucleotide

Both phosphorylated and unphosphorylated forms of nicotinamide adenine dinucleotide (NADP and NAD) are found in reactions of the metabolic network. Additionally, this coenzyme has a detailed pathway for incorporation in *M. florum*. While no transporter is specifically annotated for its import, NAD is a combination of two nucleotides joined by their phosphate groups, and it is

possible that this configuration allows it to be imported through the same transporter as nucleobases (discussed above).

Upon import, four enzymes compose this pathway. Nicotinamide is converted to nicotinate through the nicotinamidase (Mfl340, EC 3.5.1.19). A nicotinate phosphoribosyltransferase is also present in the genome (Mfl588, *pncB*). The current RefSeq annotation identified the EC number 2.4.2.11 which is obsolete according to KEGG (Kanehisa *et al*, 2017). The replacement EC number (EC 6.3.4.21) was correctly identified by both PATRIC and DETECT while COFACTOR also attributed the old EC number 2.4.2.11. An adenylyltransferase (Mfl373, *nadD*) and a NAD synthase (Mfl521, *outB*) catalyze the last two steps of this pathway leading to the formation of NAD. Also, a NAD kinase is annotated (Mfl193, *ppnk*), which supports the presence and utilization of NADP in the metabolic network.

Folate

A major pathway present in *M. florum* is the formation of folate and derivatives. A folate specific transporter is annotated in PATRIC (Mfl061 or Mfl086). A dihydrofolate reductase (Mfl383, *folA*), enables its entry into the folate pathway. Key metabolic reactions involved in the folate pathway include: the thymidylate synthase (Mfl419), producing dTMP from dUMP; the glycine hydroxymethyltransferase (Mfl106), reversibly producing serine from glycine; and the Methionyl-tRNA formyltransferase (Mfl409), producing formylmethionine, essential to initiate the translation of proteins.

Coenzyme A

In the metabolic network, Coenzyme A (CoA) is used in the biosynthesis of lipids to activate the apo-Acyl-carrier protein and in the PDH complex. Metabolically speaking, this coenzyme is therefore essential. Nevertheless, its import and synthesis remain to be characterized in

M. florum. Indeed, no transport reaction could be found that imports CoA specifically and a single enzyme, diphosphoCOA kinase (Mfl281), is annotated.

Lipoate

Lipoate is present in the PDH complex where a lipoyl-adenylate protein ligase (Mfl038) is present. The import of that coenzyme is absent as well as a potential pathway to its synthesis.

Thiamine

A thiamine diphosphokinase (Mfl224) is annotated in PATRIC. A consistent EC number (EC 2.7.6.2) was attributed to this gene by both PATRIC and COFACTOR, which is interesting since RefSeq identified it as a “hypothetical protein”. The presence of thiamine in the network is therefore supported by this annotation.

Riboflavin

A rather complete pathway leads to the formation of flavin adenine dinucleotide (FAD) in *M. florum*. This pathway includes an annotated transporter for riboflavin (Mfl576). The two following reactions are catalyzed by genes with two different EC numbers. For both Mfl283 and Mfl334 genes, PATRIC and RefSeq annotations suggest two reactions: riboflavin kinase (EC 2.7.1.26) and FAD synthase (EC 2.7.7.2).

Polyamines

Spermidine and putrescine have annotated transporters in *M. florum*. One is a spermidine/putrescine ABC transporter composed of three genes (Mfl509, Mfl510, and Mfl511). The other is a putrescine/ornithine APC transporter (Mfl664).

Minerals

The import of minerals in the metabolic network was first considered based on the known annotation. The manual curation of the genome identified inorganic phosphate, magnesium, cobalt, zinc, potassium, and sodium as potentially imported ions. Some key minerals were not gene-associated but nevertheless included in the model since they represent universally essential cofactors in prokaryotes (Xavier *et al*, 2017).

The import of inorganic phosphate is also annotated in JCVI-syn3A (Breuer *et al*, 2019) and was associated with three genes in *M. florum* (Mfl233, Mfl234 and Mfl235). Two EC numbers (EC 3.6.3.27 or 3.6.3.33) could be identified for one of these genes (Mfl235), the ATP-binding protein of the complex. Interestingly, this three-gene cluster has a transcriptional regulator right next to it, suggesting an operon-type regulation and an important feature of *M. florum*'s metabolism.

Magnesium is essential for the polymerization of nucleic acids and a specific ATPase transporter is present to ensure its import (Mfl496). Other genes are also linked to its transport through the cell membrane (Mfl217 and Mfl356). These transporters nevertheless seem to serve a more general purpose of large cation import/export, as revealed by their annotation (Mfl217, Mg/Co/Ni transporter MgtE, CBS domain-containing; Mfl356, Lead, cadmium, zinc, and mercury transporting ATPase (EC 3.6.3.3 and EC 3.6.3.5), copper-translocating P-type ATPase (EC 3.6.3.4). Other transporters responsible for the evacuation of metals are present in the *M. florum* annotation, namely the energy-coupled factor (ECF) complex (Mfl152, Mfl153, Mfl154), which corresponds to the *cbiO* transport protein of *Salmonella paratyphi A*.

Finally, potassium and sodium may be imported through a three-gene complex (Mfl164, Mfl165 and Mfl166) that are annotated as a K+, Na+ uptake protein integral membrane subunit, the *trkA* gene, and the *trkH* gene.

3. *M. florum* growth medium

The most common culture medium of *M. florum* is the ATCC 1161, a complex mixture of heart infusion broth, horse serum (HS), and YE. To test *M. florum* metabolic capabilities, we seek to replace these undefined components by a completely defined cell culture medium. We found that supplementing the commercial CMRL-1066 medium base with 0.313% HS and 0.02% YE, referred to as CSY, allowed significant growth only when a sugar source was also provided (Appendix Figure S3). Converting all medium components to BiGG identifiers (Norsigian *et al*, 2020) allowed comparing the composition of CMRL-1066 to metabolites in the reconstruction. The metabolic reconstruction provided 84 transport reactions and extracellular metabolites. To simulate growth on CSY, the *in silico* minimal medium was defined using the COBRApy toolbox (Ebrahim *et al*, 2013) (Appendix Table S2). Of the 55 components included in CMRL-1066, 36 were present in the original extracellular metabolites and 19 were missing. The missing components were evaluated individually:

- **Trans-4-hydroxy-proline:** hydroxyproline is a component of collagen. This metabolite is present in *Saccharomyces cerevisiae* where a hydroxyproline reductase is present (King *et al*, 2016). This reaction is absent from *M. florum*. Hydroxyproline is not likely to be necessary for *M. florum* growth and was therefore not added to the model.
- **4-aminobenzoate:** aminobenzoate can be converted into dihydropteroate (EC 2.5.1.15). This EC number is absent in *M. florum* L1 (see Datasets EV3 and EV4). It is not impossible that 4-aminobenzoate is used to produce folate in *M. florum,* but not enough evidence is present to add the compound to the medium.
- **Biotin:** Biotin has been reported to be an essential cofactor in bacteria but is only found in pathways absent in *M. florum* (Salaemae *et al*, 2016). These pathways include fatty acid biosynthesis, replenishment of the TCA cycle and amino acid metabolism. Since

M. florum does not contain a TCA cycle, nor any elaborate fatty acid or amino acid biosynthesis pathways, this vitamin is therefore not likely to be used.

- **Thiamin diphosphate:** a complete pathway with a specific transporter is annotated for thiamin in *M. florum*. This coenzyme is used by the PDH complex, an enzyme essential for the production of acetate in the presence of oxygen. Thiamin diphosphate is likely generated from intracellular thiamin imported from the growth medium.
- **Deoxyadenosine, deoxyguanosine, deoxycytidine:** deoxynucleosides present in the medium may be imported by *M. florum* but this hypothesis needs to be validated with a completely defined medium. The current model allows the import of guanosine, guanine, uracil, and thymidine.
- **Flavin adenosine dinucleotide (FAD):** the precursor to FAD, riboflavin, is present in CMRL-1066 and both the transporter and pathways for the production of FAD from riboflavin are present in *M. florum*. This metabolite is not likely to be used by *M. florum*.
- **Nicotinamide adenine dinucleotide (Phosphate) (NAD, NADP) and nicotinic acid:** the precursor of these three components, nicotinamide, is present in CMRL-1066 and both the transporter and pathways for the production of NAD from nicotinamide are present in *M. florum*. These three components are likely to be redundant with the presence of nicotinamide.
- **Pantothenate:** this coenzyme is involved in the synthesis of CoA, which is already present in CMRL-1066, and that *M. florum* is incapable of synthesizing. This component is likely to be non-essential for *M. florum*.
- **Pyridoxine and pyridoxal:** also known as vitamin B6, pyridoxine was not directly involved as a coenzyme in any metabolic reactions. Whether or not *M. florum* requires this coenzyme for growth should be assessed upon the elaboration of a completely defined medium.
- **Sulfate:** cysteine desulfurase seems to be taking the role of providing the cell with sulfur,

an essential metabolite.

- **Glutathione:** as a tripeptide, this medium component could be imported by the peptide importer system. Given its role as an antioxidant, its import could reduce the susceptibility to oxidative stress in *M. florum* when added to the medium. This hypothesis could be tested by growth assays under oxidative stress with or without glutathione.
- **Glucuronate:** this monosaccharide is involved in proteoglycan synthesis in many species. It is likely that its import could be done by one of the sugar importers and it could potentially contribute to the synthesis of the *M. florum* glycans.
- **Cholesterol:** helps to solubilize FFA and facilitate their import.
- **Tween 80:** also helps to solubilize FFA and facilitate their import.

4. Conversion into a mathematical format

4.1. Biomass objective function

The biomass objective function (BOF) represents the sum of all metabolic goals of an organism in a given environment. In order to be representative of the cellular state, the biomass function should be derived from experimental measurements. Previous work yielded the detailed composition of *M. florum* biomass (Matteau *et al*, 2020). Along this data, the BOFdat software (Lachance *et al*, 2019) was used to determine the biomass precursors to include to the BOF and their respective stoichiometric coefficients (see Figure 5A). Genomic (DNA), transcriptomic (RNA) and proteomic (proteins) data along with macromolecular weight fractions (MWF) for each category were used as input to determine stoichiometric coefficients using the Step1 of BOFdat (Appendix Table S3).

The Step2 of BOFdat identified 16 coenzymes and cofactors to be added to the biomass. Ions that are commonly found in bacteria are also identified in the reconstruction. 12 ions were identified in this step (calcium, manganese, cobalt, molybdate, chloride, sodium, ammonium, zinc, potassium, nickel, magnesium and hydrogen). The only coenzyme identified was nicotinamide and its derivatives: oxidized and reduced versions of the phosphorylated and non-phosphorylated forms (Appendix Table S3 and Figure 5A).

Lipids and glycans were not added to the equation by the first and second step. The decision was made to forgo their addition in these steps since the experimental data required curation. Their inclusion, along with other metabolites, was left to the unbiased genetic algorithm performed in BOFdat Step3 (Figure 5A, Table 2, and Appendix Figure S7). Accordingly, two lipids were identified (phosphatidylcholine, phosphatidylglycerophosphate). Supporting the evidence for the presence of phosphatidylcholine in the membrane was the identification of the phosphorylated

version of choline. The Acyl-carrier protein was also added given its importance for the synthesis of lipids and its ubiquitous presence in prokaryotes. The capsular polysaccharide metabolite formulated during the reconstruction was also added during this step (Appendix Table S3).

Interestingly, S-adenosyl methionine was identified in BOFdat Step3. This metabolite is a common co-substrate involved in the transfer of methyl groups and is excessively important in many organisms. Consistent with this identification, methyltetrahydrofolate and sulfur were also identified and added during Step3.

The polyamines spermidine and putrescine were added during BOFdat Step3. The exact function of these metabolites is not precisely known in prokaryotes, but they are found widely across species. Putrescine was nevertheless not found in CMRL-1066. Depriving *M. florum* from either of these polyamines in a completely defined medium could shed light on their function in prokaryotes.

Finally, cytidine and adenosine were identified by BOFdat Step3, which can probably be attributed to the essentiality of the genes that make these specific metabolites. This likely means that some routes that were proposed in the nucleotide salvage pathway are not actually possible *in vitro*.

4.2. Sensitivity analysis

The main carbohydrate provided in ATCC 1161 medium is sucrose. Hence, when grown in CSY medium, *M. florum* was also provided with sucrose and its specific uptake rate was measured with high-performance liquid chromatography (HPLC) (Figure 4E, Appendix Figures S4, S5AB and S6AB). The obtained value was -5.26 mmol per gram of *M. florum* dry weight per hour ($gDW^{-1}\cdot hr^{-1}$), which is similar to previously published results for glucose uptake rate in *M. pneumoniae* (7.37 $mmol\cdot gDW^{-1}\cdot hr^{-1}$) and *M. gallisepticum* (16.53 $mmol\cdot gDW^{-1}\cdot hr^{-1}$) (Wodke *et*

al, 2013; Bautista *et al*, 2013) (Table 3). Nevertheless, to our knowledge, the sucrose uptake rate calculated here is a first amongst Mollicutes. This value is slightly lower than values previously observed for *E. coli*, which ranged between 7.01 and 14.10 mmol·gDW^{-1}·hr^{-1} following adaptive laboratory evolution (Mohamed *et al*, 2019).

The secretion rates were obtained for both lactate and acetate, the two possible fermentation products in *M. florum* (Figures 2 and 4F, Appendix Figure S5C and S6C). The cumulative value for both products was 8.69 mmol·gDW^{-1}·hr^{-1}, which is exactly in the range of acetate secretion rates in *E. coli* (4.2 to 15.9 mmol·gDW^{-1}·hr^{-1}), slightly higher than *M. pneumoniae* (6.93 mmol·gDW^{-1}·hr^{-1}), and lower than *M. gallisepticum* (10.29 mmol·gDW^{-1}·hr^{-1}) (Table 3).

While an acetate secretion rate was measured in *M. pneumoniae*, a lactate secretion rate was instead measured in *M. gallisepticum*. The metabolic reconstruction of *M. florum* shows that both secretion pathways are complete and should be functional. Since our current experimental setup did not allow the direct measurement of each secretion product, the exact secretion rates still remain hypothetical. The model can nevertheless reveal some interesting trade-offs around the production of lactate and acetate. The path to lactate secretion is rather simple with a single enzyme, the LDH (Mfl596), converting pyruvate into lactate. The secretion of acetate is more intricate and involves a complete operon composed of the lipoate-ATP adenylate transferase (Mfl038), the complete PDH complex (Mfl039, *pdhA*; Mfl040, *pdhB*; Mfl041, *pdhC*; and Mfl042, *pdhD*), a phosphotransacetylase (Mfl043, *pta*), and an acetate kinase (Mfl044, *ackA*). Contrary to lactate production, the path to acetate releases CO_2 as a metabolic waste, yields one ATP but reduces one NAD molecule into NADH. While the production of one molecule of ATP seems profitable for the cell, the one NADH molecule must be re-oxidized to make this process sustainable. One key reaction involved in this process is the NADH oxidase (Mfl037, *nox*). This enzyme uses molecular oxygen (O_2) to convert NADH back to NAD. This reaction exists in two

forms: H_2O producing or H_2O_2 producing. While producing water molecules is not harmful for the cell, hydrogen peroxide is a toxic waste that needs to be eliminated. While this task could be achieved by the L-methionine S oxide reductase (Mfl050, *msrA*), the specificity to H_2O_2 is not confirmed. The final model therefore uses the NOX2 reaction (BiGG identifier), which produces water instead of hydrogen peroxide. Detecting the production of hydrogen peroxide by *M. florum* could shed light on this process.

The acetate production can be probed using *i*JL208. In the final version of the model, the lactate secretion was favored since the expression of the LDH was much higher than the PDH complex (Matteau *et al*, 2020). To favor the production of lactate, restrictive bounds were applied to key reactions. The upper bound to the NOX reaction was fixed at 5 $mmol \cdot gDW^{-1} \cdot hr^{-1}$. This limits the amount of oxygen that can be used to oxidize NADH back to NAD which can be used in the glycolysis. Since *M. florum* is a facultative aerobe, the logical decision was to limit the impact of oxygen on its growth phenotype. Considering that the NADH oxidase does not have an unlimited capacity was one option, the other was to reduce the possibility for oxygen import. This could be done by reducing the lower bound of its exchange reaction (EX_o2_e).

To probe the production range of acetate, the bounds can be changed on these critical reactions. Providing equal lower and upper bounds on the secretion of lactate and acetate, here 0 and 10, releases the experimental constraints. With these bounds applied, increasing the limit oxygen uptake rate and the upper bound on the NADH oxidase reaction eventually results in a favorable utilization of the acetate secretion pathway. In this small case study, this was observed when the bounds were at 25, which is >1.5 times the upper bound on the secretion rates or approximately >5 times the sucrose uptake rate.

These model predictions stating that a very high amount of oxygen is required to efficiently

produce acetate are consistent with the expression levels of *ldh* and *pdh* genes which suggest a higher production of lactate. The settings implemented in the final version of *i*JL208 ensured that the ATP synthase pump was essential as observed experimentally, which also supported the final choice of constraints.

5. Validation of model phenotypic predictions

5.1. Carbohydrates utilization

Reducing the concentration of rich undefined components in the medium revealed a clear difference between sucrose supplemented medium and a no-sugar control (Figure 4A and Appendix Figure S3), further enabling to validate the assimilation of 14 different carbohydrates by *M. florum.* Upon comparison with model predictions, eight no-growth and four growth phenotypes were correctly predicted (Figure 6A and Figure EV1). Two additional sugars, mannose and maltose, were found to be utilized by *M. florum* but had not been predicted by the model.

To recover those phenotypes, the alternate carbon metabolism of *M. florum* was studied, seeking enzymes that would likely carry a promiscuous activity. The three-dimensional structures reconstructed with I-TASSER were leveraged for that task (Figure 1C and Dataset EV2). While the specificity of transporters could not be addressed with this method, downstream enzymes allowing the catabolism of mannose and maltose could be compared with the PDB using the FATCAT 2.0 server (Li *et al*, 2020). Specifically, the annotation of three enzymes (Mfl120, Mfl254 and Mfl499) involved in the assimilation of glucose and trehalose were considered. Using the same approach, the specificity of two aldolases (Mfl121 and Mfl639) were assessed to recover the expression phenotype of enzymes of the PPP (Figure EV2 and Appendix Table S4).

The structural similarity between maltose and trehalose suggested they could use the same route into glycolysis. While the promiscuity of the transporter used to import maltose could not be tested *in silico*, it was hypothesized that the trehalose hydrolase (Mfl499) could also hydrolyze maltose. To generate a 3D structure for Mfl499, I-TASSER used the *Bacillus sp.* α-glucosidase BspAG13_31A (PDB: 5zcc) as a template given the similarity of both sequences. This template structure was shown to have a high-specificity to α-(1-4)-glucosidic linkage (Auiewiriyanukul *et al*,

2018). The reconstructed structure was compared to the template used by I-TASSER with FATCAT 2.0 ($p = 0.00$, Figures 6B and EV2). The sequence and structural similarity with an enzyme capable of acting on both maltose and trehalose supports the hypothesis that Mfl499 is involved in maltose assimilation. The addition of both the promiscuous transport and cleavage reactions were sufficient to provide a growth prediction on maltose.

The capability of *M. florum* to metabolize mannose could be explained if the glucose-6-phosphate (G6P) isomerase, PGI, (Mfl254) was able to convert mannose-6-phosphate (M6P) into fructose-6-phosphate (F6P), hereby entering glycolysis. The reconstructed structure of the *M. florum* PGI was compared to that of *Pyrobaculum aerophilum* (Swan *et al*, 2004) (PDB:1TZB), known for its capability of converting either G6P or M6P into F6P. The structural similarity between these enzymes ($p = 8.68e\text{-}12$, Figure EV2) was consistent with this hypothesis and the model was modified accordingly.

The utilization of mannose was also studied in the context of glycan synthesis. Gas chromatography previously revealed the presence of both glucose and mannose in the CPS of *M. florum* (Matteau *et al*, 2020). The presence of a phosphomannomutase, PMM, (Mfl120) in the annotation suggested the conversion of M6P in mannose-1-phosphate (M1P), a necessary precursor for glycan synthesis (Bertin *et al*, 2015). The template used by I-TASSER for the reconstruction of the 3D structure of Mfl120 was the PMM/PGM structure from *Pseudomonas aeruginosa* (PDB:1K35). In this organism, the enzyme is necessary for the production of exopolysaccharides (Regni *et al*, 2002) with G6P and M6P entering the glycan synthesis pathway through the same enzyme. Given the structural similarity of the *M. florum* and *P. aeruginosa* enzymes (Figure EV2), the promiscuous mutase reaction catalyzed by Mfl120 was added to the model and was sufficient to formulate a positive growth prediction for mannose.

5.2. Validation with proteomic and transcriptomic data

Gene expression

Flux balance analysis enables the prediction of other phenotypes such as flux states and gene essentiality. These predictions can be used along with experimental data to improve the model quality (Thiele & Palsson, 2010). Genome-wide expression (Matteau *et al*, 2020) and transposon insertion (Tn-Seq) (Baby *et al*, 2018) datasets available for *M. florum* were used as a reference for the validation of model predictions. Gene expression was compared to the model predicted flux states by converting both datasets to binary “on” or “off” values. The set of expressed genes was defined by finding the thresholds that would provide the best match between transcriptomic and proteomic data while maximizing the number of expressed genes (Appendix Figure S8 and Dataset EV5). At the selected thresholds, 531 genes had a consistent signal in both proteomic and transcriptomic data while 145 had mixed signals (e.g., proteomic “on” and transcriptomic “off”). Only the genes for which datasets were consistent with each other were used for comparison with the model. This set contains 423 expressed and 108 silent genes (Appendix Figure S8). Weiner and colleagues (Weiner *et al*, 2003) previously reported that, using mRNA expression in *M. pneumoniae*, 564 of the 676 genes were considered expressed. While this number is higher than the 423 we report in *M. florum*, it is quite comparable if we consider only the number of fragments per kilobase per million of mapped reads (FPKM) generated from the transcriptomic data, resulting in 525 expressed genes (mean FPKM = 1221.0) at the selected threshold (FPKM = 168.0). Combining both datasets hence provides a more conservative observation.

The flux state through the metabolic network was obtained by optimizing the production of biomass using parsimonious flux balance analysis (pFBA), a version of FBA that allows the generation of a unique flux state prediction through minimization of enzyme usage (Lewis *et al*,

2010). This method is best suited for the comparison of predicted fluxes to gene expression (Machado & Herrgård, 2014). A reaction flux was defined as active when the predicted value exceeded the numerical error (1e-8), and the flux was attributed to every gene that could catalyze the reaction via the gene-reaction rule. The comparison of binary flux predictions and observed expression was performed on the subset of model genes (173/208) for which proteomic and transcriptomic data showed no discrepancy.

Gene essentiality

Single gene essentiality was reported previously (Baby *et al*, 2018) where ~290 *M. florum* genes were proposed to be essential, which was considerably inferior to the 382/482 essential genes reported by Glass and colleagues in *M. genitalium* (Glass *et al*, 2006) and the 473 genes in the minimal cell JCVI-syn3.0 (Hutchison *et al*, 2016). This number was revisited by including both growth data for mutants with transposon insertion (Dataset EV5) as well as the relative position of the insertion within the gene (Figure EV3). The latter assumes that an insertion in the final 20% section of the gene would not hamper its activity, a method that proved useful in the design of JCVI-syn3.0 (Hutchison *et al*, 2016). The number of essential genes was therefore raised to 362 (Figure EV3). The fact that the single gene essentiality is not equal to the number of genes in JCVI-syn3.0 indicates that there is still redundancy in *M. florum* that could be removed by genome reduction. Single-gene knockout growth simulations were performed on every gene in the model, hereby generating a prediction of essentiality that can be compared to the experimental transposon insertion dataset.

Comparing with model predictions

Comparing the model predictions with experimental data initially revealed erroneous predictions of the model that were manually addressed. True false negatives (TFN) were defined as genes simultaneously expressed and essential while no flux or essentiality was predicted. Eight TFN

were identified. A single true false positive (TFP) was found, which had both flux and essentiality prediction but no observed expression nor essentiality. Curating these genes allowed increasing the model accuracy in the prediction of gene expression from 74.25% to 78.03% and essentiality from 74.52% to 76.92%. It is noteworthy that solving more than the TFP and TFN would mean fitting the model specifically to either essentiality of expression datasets.

The accuracy of the *i*JL208 model is slightly lower than other Mollicutes' models (*M. genitalium* (Suthers *et al*, 2009), 79% initial and 87% after GrowMatch (Kumar & Maranas, 2009); *M. pneumoniae*, 86% (Wodke *et al*, 2013)). Two factors may be responsible for that reduced accuracy. First, the number of genes included in the *M. florum* model is higher and some of these genes have a presumptive annotation. The choice of including more genes in the model was compensated by the attribution of a level of confidence for the added genes. Second, *M. florum* has not been as characterized from a biochemical standpoint than these other Mollicutes. The development of this model provides a systematic approach to target the gaps in the current knowledge and should bolster the efforts towards characterization of *M. florum*'s molecular functions.

Curating True False Negatives (TFN)

Two cases could be solved by the addition of a forced flux (DHAK, Mfl229 and dUPTase, Mfl257), two by the addition of new components to the biomass (RBFK, Mfl334 and ACPS, Mfl384), while the other four had missing information. It was the case for Mfl558, which is annotated as a chitin deacetylase. Chitin is most likely absent from the growth medium in which the experimental data was generated, hence the reported expression and essentiality likely indicates that this enzyme was mis-annotated. The specificity of the inosine-5-monophosphate dehydrogenase (Mfl343) should also be addressed as it could not be explained in the current metabolic network. Finally, the necessity of the CTP synthase (Mfl648) suggested that the exchange of amino groups within

M. florum occurred primarily through exchanges in the glutamine/glutamate pool rather than through the import of ammonium from the medium but insufficient information was found in the literature to support that hypothesis.

Solving false negatives required the addition of specific constraint(s). The dUTPase (Mfl257) and dihydroxyacetone kinase (Mfl229) are simple examples of such cases (Appendix Figure S9A). In the first case, the accidental production of dUTP by the cell was mimicked by forcing a flux through the PYK10 reaction which produces it from dUDP. In turn, this forces the activity of Mfl257 to produce dUMP and a pyrophosphate. The second case represents a similar cellular situation where the highly reactive molecule dihydroxyacetone phosphate spontaneously loses its phosphate yielding dihydroxyacetone, a toxic molecule for the cell. A forced flux through this spontaneous reaction resolved the discrepancy, making Mfl229 carrying flux and being essential (Appendix Figure S9B).

A more complicated case is observed for the ribulose-5-phosphate epimerase (Mfl223). Activating it in the model requires forcing flux through the PPP. In many Mollicutes, this pathway is incomplete (Breuer *et al*, 2019; Wodke *et al*, 2013; Suthers *et al*, 2009; Miles, 1992), often because no gene can be attributed to the transaldolase reaction (TALA). The structures of two aldolases (Mfl121 and Mfl639) were compared against the PDB 90 using FATCAT 2.0 (Li *et al*, 2020). For both structures, a significant similarity was observed against the A chain of the transaldolase of *Thermotoga maritima* (1vpxA; TM0295, Mfl121 p-value: 5.96e-10; Mfl639 p-value: 2.51e-9).

Adding the TALA reaction enables flux through the PPP but does not force it since active uptake of ribose circumvents its need. The non-essentiality/non-expression of both ribose kinase (Mfl642, *rbsK*) and ribose ABC transporter (Mfl666, Mfl667, Mfl668, Mfl669) suggests that ribose was

altogether absent from the ATCC 1161 growth medium in which the datasets were generated. Ribose was therefore removed from the *in silico* medium, resulting in flux through the PPP and increased prediction accuracy for expression.

Curating True False Positive (TFP)

The path for synthesis of nicotinamide dinucleotide in *M. florum* was discussed above. The presence of a nicotinamidase (Mfl340) suggested the import of nicotinamide from the medium. Nevertheless, experimental data revealed that this enzyme is both non-essential and non-expressed, suggesting that the downstream metabolite, nicotinate, may be imported instead. Adding this metabolite to the *in silico* media as well as an import reaction avoids the need for Mfl340, recapitulating experimental observations (Appendix Figure S9C).

6. Model-driven prediction of a minimal genome

Varying the growth rate results in different genome reduction scenarios

Together with experimental gene essentiality and the transcription unit architecture, *i*JL208 was used to formulate a minimal genome prediction. Using the MinGenome algorithm (Wang & Maranas, 2018), genome reduction scenarios were generated at different growth rates (Appendix Figure S10). This was made possible because MinGenome attempts to find the largest possible deletion in the genome without breaking the established constraints. The constraints imposed include the impossibility to delete an essential or its associated promoter and ensuring the feasibility of the GEM. The model's objective and value are therefore fixed. If a gene deletion prevents the model from solving at this specific growth rate, then the deletion is not possible.

Varying the minimum growth rate imposed as a constraint on the optimization problem formulated with MinGenome enables the deletion of genes that could hamper the growth rate without being completely lethal. While an array of growth rates was tested, only three different genome reduction scenarios were obtained. The similarity of the resulting genomes to JCVI-syn3.0 were assessed for each growth rate constraint imposed. The genomes formulated with a lower minimum growth rate constraint were more similar to JCVI-syn3.0 (Appendix Figure S10). The final genome size of the lower growth rate constraint scenarios was also smaller than higher ones.

In all cases, no more genes could be deleted after 80 deletions. A minimal size was reached at 60% of the optimal growth rate, yielding a 562 kbp genome containing 563 genes and corresponding to a ~30% reduction from the initial *M. florum* L1 genome. In size, this minimal genome scenario lies between the JCVI-syn3.0 inspired (470 kbp, 409 genes) and the core genome of *M. florum* (644 kbp, 585 genes) suggested by Baby and colleagues (Baby *et al*, 2018). While this model-driven prediction does not reduce the number of genes beyond that of

JCVI-syn3.0, a reduction of 30% in genome size is a level that has been reached experimentally in different species. In fact, the *B. subtilis* genome was trimmed by 36% and yielded a functional genome (Reuß *et al*, 2017) with growth rates similar to the wild-type strain. To our knowledge, the smallest *E. coli* genome allowing robust growth is that of DGF-298 (Hirokawa *et al*, 2013). At 2.98 Mbp, this genome represents a 34.4% reduction compared to the original *E. coli* K-12 substr. W3110 (Westphal *et al*, 2016).*B. subtilis* and *E. coli* reduced genome strains yielded robust cells with growth rates similar to their parental strain. This was not the case for JCVI-syn3.0, which was originally reported to have a lower growth rate and an altered morphology (Hutchison *et al*, 2016). In order to produce a more robust and functional cell usable in the laboratory, 19 genes were added back into the original JCVI-syn3.0, generating a 493 genes bacterium called JCVI-syn3A (Breuer *et al*, 2019). Our current prediction of a minimal gene set is therefore 70 genes above JCVI-syn3A (563 genes).

Functional analysis of the reduced genome

The functional categories in which the deleted/retained proteins were analyzed using the KEGG ontology (Figure 7C and Figure EV4). Interestingly, the largest portion of *loci* considered for deletion belonged to the unmapped category (81). Reducing the number of unknown components is a key argument justifying research efforts in minimal cells. Identifying these non-essential hypothetical proteins was therefore crucial for further experimental efforts to reduce the genome of *M. florum*.

Next, we compared the number of genes in each KEGG functional category identified as deletion targets to the genes kept in the reduced genome scenario. Interestingly, the main category affected by deletions were uncharacterized proteins (“Not mapped”) with 81 proteins (~56% of all deleted proteins), and a small fraction of those (16) had homologs in JCVI-syn3.0 (Figure 7CD and Figure EV4). With 191 proteins out of 535, the proportion of uncharacterized proteins retained

in the *M. florum* reduced genome scenario (~36%) is also very similar to the reported proportion in JCVI-syn3.0 (149/438, 34%).

The second KEGG category with the highest number of deletions was “Metabolism”, with 34 proteins removed (Figure 7CD and Figure EV4). 155 proteins of this category remained in the reduced genome and 54 of those were not homologous to JCVI-syn3.0. Specifically, proteins deleted in *M. florum* but present in JCVI-syn3.0 were mostly found in the transport sub-category (Mfl019, Mfl234, Mfl533, Mfl534) and are annotated as ABC transporters. In accordance with our experimental data and *i*JL208, both the glutamine ABC transporter (Mfl019) and the phosphate ABC transporter (Mfl234) were not essential in *M. florum* since other routes exist for their import. Mfl533 and Mfl534 were annotated as lipid A export proteins (*msbA*) but no evidence supports the presence of this metabolite in *M. florum*. Since the lipid module was amongst the least characterized and given its status in our prediction, these genes represent top priorities for further biochemical characterization. Comparing the deleted proteins with the remaining ones in *i*JL208 revealed that gene redundancy in the sucrose PTS importer allowed to keep this function in the reduced genome. Contrarily, the trehalose PTS was completely removed suggesting that alternate versions of a minimal gene set for *M. florum* may have different auxotrophies.

Finally, the ”Genetic information processing” (GIP) category was the least affected by deletions and contained the highest number of proteins in the reduced genome scenario (Figure 7CD and Figure EV4). This category also contains the highest proportion of proteins shared with JCVI-syn3.0 (~89%).

We provide here a detailed analysis of the composition of the reduced genome by functional category. Detailed information is available in Dataset EV6:

Not mapped:

191 not mapped proteins retained in the reduced genome with 106 proteins specific to *M. florum* (absent from either JCVI-syn1.0 and JCVI-syn3.0).

Metabolism:

The metabolism category is composed of 12 sub-categories, three of which exclusively contain genes that have homologs in JCVI-syn3.0: ATP synthase, amino acid metabolism, and secretion system. To evaluate the possibility for further reduction or potential alternative genome reduction scenarios, we detail the composition of the remaining nine metabolism sub-categories.

- **Transport:**

  This sub-category contains 33 proteins with eight *M. florum* specific proteins, 18 homologs to JCVI-syn3.0, and seven JCVI-syn1.0 homologs. The main features shared with JCVI-syn3.0 in this category are the following: oligopeptide ABC transporter, cobalt transporter, K+/NA+ uptake protein, phosphate ABC transporter, spermidine/putrescine ABC transporter, phosphonate ABC transporter (half retained) and ribose ABC transporter.

  The transport proteins that were not retained in JCVI-syn3.0 are the following: Amino acid transporter permease (Mfl184), Mg/Co/Ni transporter, formate/nitrate transporter, Mg2+ transport, and xanthine/uracil permease.

- **PTS:**

  This sub-category contains five proteins, with one specific to *M. florum*, three having homologs in JCVI-syn3.0, and one homolog to JCVI-syn1.0. The retained functions in JCVI-syn3.0 are: phosphoenolpyruvate-protein phosphotransferase, PTS phosphohistidine, and the glucose specific PTS transporter subunit.

The PTS related proteins that were retained in the reduced genome but not shared with JCVI-syn3.0 are: sucrose specific PTS component, and fructose specific PTS component.

- **Glycolysis and carbohydrate metabolism:**

  This sub-category contains 27 proteins, with eight specific to *M. florum*, 15 having homologs in JCVI-syn3.0, and four homologs in JCVI-syn1.0. The retained functions in JCVI-syn3.0 are: PDH, phosphomannomutase, pyruvate kinase, as well as all glycolysis enzymes and secretion routes for both lactate and acetate.

  Glycolysis and carbohydrate metabolism related proteins that were retained in the reduced genome but not shared with JCVI-syn3.0 are: all beta-glucosidases, both E1 subunits of the PDH, a sucrose-6-phosphate hydrolase, a fructokinase and a 1-phosphofructokinase.

- **Pentose phosphate metabolism:**

  This sub-category contains eight proteins, with three specific to *M. florum*, five having homologs in JCVI-syn3.0, and no homologs in JCVI-syn1.0. The retained functions in JCVI-syn3.0 are: phosphoribosylpyrophosphate synthetase, ribose-5-phosphate isomerase, transketolase, one of the two 2-deoxyribose-5-phosphate aldolase and one of the two pentose-5-phosphate epimerase.

  Pentose phosphate metabolism related proteins that were retained in the reduced genome but not shared with JCVI-syn3.0 are: one of the two 2-deoxyribose-5-phosphate aldolase, one of the two pentose-5-phosphate epimerase, as well as the ribokinase.

- **Cofactor biosynthesis:**

This sub-category contains 16 proteins, with two specific to *M. florum*, seven having homologs in JCVI-syn3.0, and seven homologs in JCVI-syn1.0. The retained functions in JCVI-syn3.0 are: cytosol aminopeptidase (duplication or complex), riboflavin kinase (duplication), nicotinate-nucleotide adenylyltransferase, nicotinate phosphoribosyltransferase, NAD kinase, holo-ACP synthase, folyl-polyglutamtate synthetase.

Cofactor biosynthesis related proteins that were retained in the reduced genome but not shared with JCVI-syn3.0 are: lipoate protein ligase, dephospho-CoA kinase, nicotinamidase/pyrazinamidase.

- **Purine and pyrimidine metabolism:**

  This sub-category contains 20 proteins, with two specific to *M. florum*, 14 having homologs in JCVI-syn3.0, and four homologs in JCVI-syn1.0. The retained functions in JCVI-syn3.0 are: thioredoxin reductase, phosphoribosyl transferases for uracil, adenine, and hypoxanthine-guanine, as well as kinases for adenylate, guanylate, cytidylate, thymidylate, and finally a purine-nucleoside phosphorylase.

  Purine and pyrimidine metabolism related proteins that were retained in the reduced genome but not shared with JCVI-syn3.0 are: adenylosuccinate lyase, thymidine phosphorylase, cytidine deaminase, guanosine-5-monophosphate oxidoreductase, Inositol-5-monophosphate dehydrogenase, xanthosine triphosphate pyrophosphatase.

- **Lipid metabolism:**

  This sub-category contains eight proteins, with none specific to *M. florum*, seven having homologs in JCVI-syn3.0, and one homolog to JCVI-syn1.0. The retained functions in

JCVI-syn3.0 are: glycerol-3-phosphate acetyltransferase, CDP-diglyceride synthetase, 1-acyl-sn-glycerol-3-phosphate acyltransferase, cardiolipin synthase, acyl carrier protein, phosphatidylglycerophosphate synthase.

The only Lipid metabolism related proteins retained in the reduced genome but not shared with JCVI-syn3.0 is the NAD-dependent-glycerol-3-phosphate dehydrogenase.

Genetic Information Processing:

The Genetic Information Processing general category contains 13 sub-categories, five of which contain exclusively genes that have homologs in JCVI-syn3.0: RNA polymerase, Translation factors, Sulfur relay system, Protein export, and tRNA loading and maturation. To evaluate the possibility for further reduction or potential alternative genome reduction, we detail the composition of the remaining eight Genetic Information Processing sub-categories. Since most of the proteins within this general category have homologs in JCVI-syn3.0, we only provide the detail of the discrepancies between the reduced *M. florum* genome and JCVI-syn3.0.

- **DNA repair**

  This sub-category contains 18 proteins, with one specific to *M. florum*, 11 having homologs in JCVI-syn3.0, and six homologs in JCVI-syn1.0. DNA repair related proteins that were retained in the reduced genome but not shared with JCVI-syn3.0 are: Recombination proteins (U and A, Mfl267 DNA repair/recombination protein is retained), Uracil-DNA glycosylase, DNA-3-methyladenine glycosidase, Exodeoxyribonuclease V (but VII is retained), DNA polymerase IV, and formamidopyrimidine-DNA glycosylase.

- **DNA replication and partition**

  This sub-category contains 26 proteins, with one specific to *M. florum*, 23 having homologs

in JCVI-syn3.0, and two homologs in JCVI-syn1.0. The three proteins DNA replication and partition related proteins that were retained in the reduced genome but not shared with JCVI-syn3.0 are: DNA cytosine methyltransferase (Mfl308), SepF/FtsZ-interacting protein related to cell division (Mfl391), Ribonuclease HII (EC 3.1.26.4) (Mfl537).

- **Transcription factors**

  This sub-category contains five proteins, with none specific to *M. florum*, three having homologs in JCVI-syn3.0, and two homologs in JCVI-syn1.0. The two transcription factors that were retained in the reduced genome but not shared with JCVI-syn3.0 are: an unknown transcriptional regulator and a transcriptional repressor of the fructose operon, DeoR family (consistent with metabolism).

- **Ribosome**

  The Ribosome contains 50 proteins, with a single one specific to *M. florum*, and the 49 others having homologs in JCVI-syn3.0. The single ribosomal protein that is retained in the reduced genome but not shared with JCVI-syn3.0 is the 50S ribosomal protein L33. Interestingly, this protein was also absent from JCVI-syn1.0 but was introduced when generating JCVI-syn3A.

- **Ribosome biogenesis**

  This sub-category contains 26 proteins, with one specific to *M. florum*, 23 having homologs in JCVI-syn3.0, and two homologs in JCVI-syn1.0. The three proteins that were retained in the reduced genome but not shared with JCVI-syn3.0 are all methyltransferases: 16S rRNA (cytosine(967)-C(5))-methyltransferase, 16S rRNA (uracil(1498)-N(3))-methyltransferase (EC 2.1.1.193), and RNA binding methyltransferase FtsJ like.

- **Nucleases:**

  This sub-category contains eight proteins, with none specific to *M. florum*, seven having homologs in JCVI-syn3.0, and one homolog in JCVI-syn1.0. The single protein that was retained in the reduced genome but not shared with JCVI-syn3.0 is the Mg2+ dependent DNAse.

- **Chaperones**

  This sub-category contains five proteins, with none specific to *M. florum*, three having homologs in JCVI-syn3.0, and two homologs in JCVI-syn1.0. The two proteins that were retained in the reduced genome but not shared with JCVI-syn3.0 are the hsp33 redox-regulated chaperone and the cell division trigger factor (EC 5.2.1.8).

- **Peptidases**

  This sub-category contains five proteins, with two specific to *M. florum*, three having homologs in JCVI-syn3.0, and no homologs in JCVI-syn1.0. The two proteins that were retained in the reduced genome but not shared with JCVI-syn3.0 are the intramembrane protease RasP/YluC, implicated in cell division based on FtsL cleavage and the GMP synthase [glutamine-hydrolyzing], amidotransferase subunit (EC 6.3.5.2) / GMP synthase [glutamine-hydrolyzing], ATP pyrophosphatase subunit (EC 6.3.5.2).

Appendix Figures

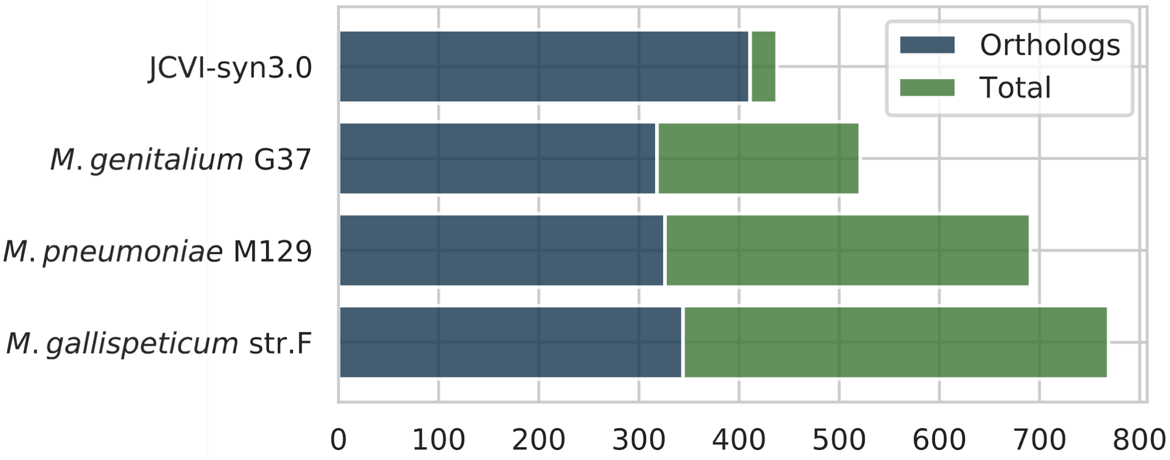

Appendix Figure S1. Orthologous proteins in other Mollicutes species with an existing metabolic model. Orthologous proteins were identified using the PATRIC proteome comparison tool. The total number of genes is presented for each species, and the number of orthologs is indicated in blue. The synthetic bacterium JCVI-syn3.0 has both the smallest protein count and the highest number of orthologs with *M. florum* L1.

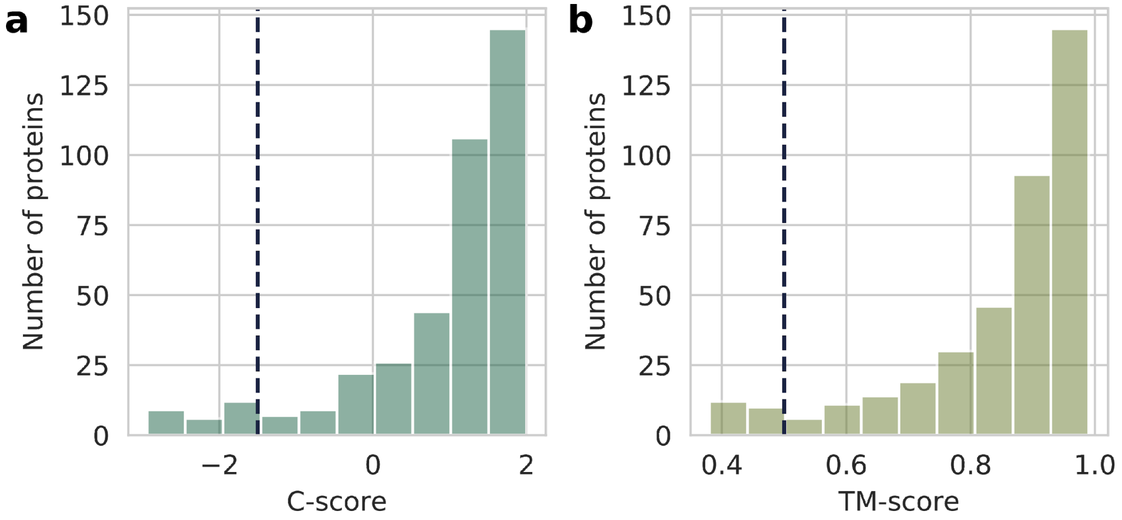

Appendix Figure S2. Distribution of the scores from the 3D protein reconstructions obtained with I-TASSER. Based on the documentation, a model structure is considered reliable if its C-score (**a**) and TM-score (**b**) are above -1.5 and 0.5, respectively. Most of the modelled proteins (361/386) had scores higher than these thresholds (dark blue dotted lines).

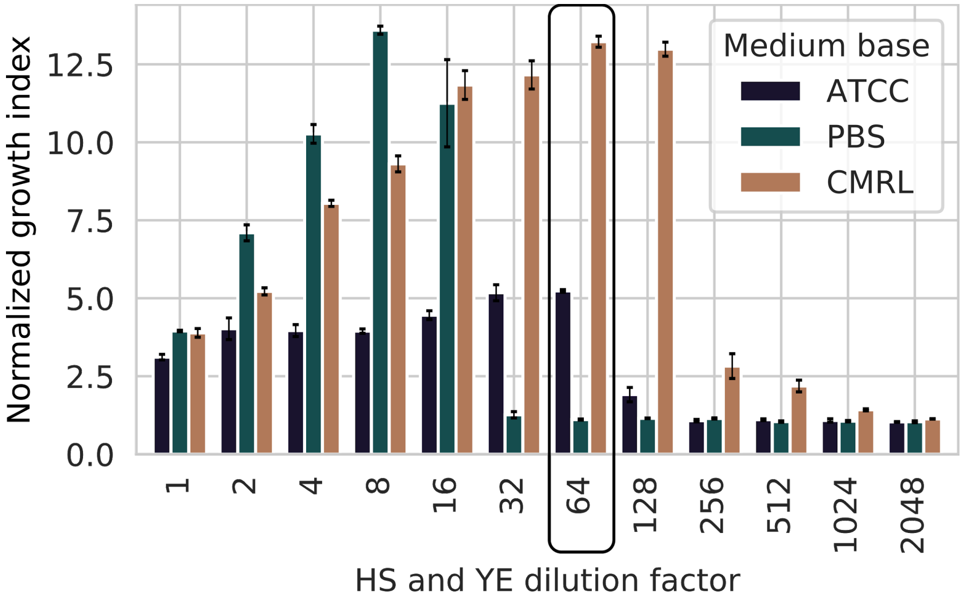

Appendix Figure S3. *M. florum* growth medium simplification. Growth medium was modified to maximize the difference in apparent growth between sugar supplemented and non-supplemented media. The normalized growth indexes (color fold change after 24 hours over a no sugar control) determined in different medium bases (ATCC 1161, CMRL-1066, and PBS 1X) supplemented with decreasing concentrations of horse serum (HS) and yeast extract (YE) are indicated. The black box shows maximum difference in color fold change for the highest depletion in HS and YE (0.313% HS and 0.02% YE) when using a CMRL-1066 medium base (CSY medium), corresponding to a 64-fold dilution factor over the concentrations found in ATCC 1161 (20% HS/1.35% YE). Bars and error bars indicate the mean and standard deviation calculated from technical triplicate.

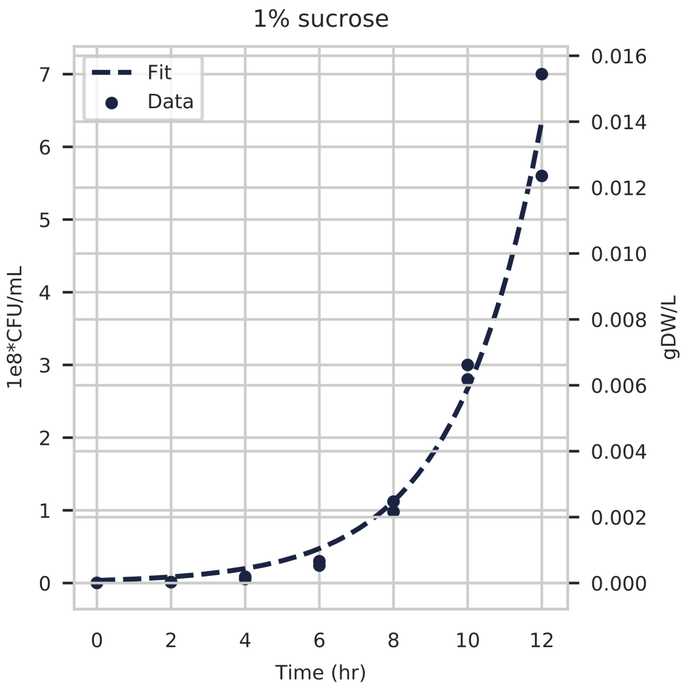

Appendix Figure S4. Biomass concentration over time of a *M. florum* culture growing in CSY medium with 1% sucrose. Biomass was measured using colony forming units (CFU/ml; left axis) and converted to grams of dry weight (gDW/L; right axis). CFU/ml quantifications were performed in technical duplicate. All data points are shown. A simple exponential growth fit is also displayed (dotted line).

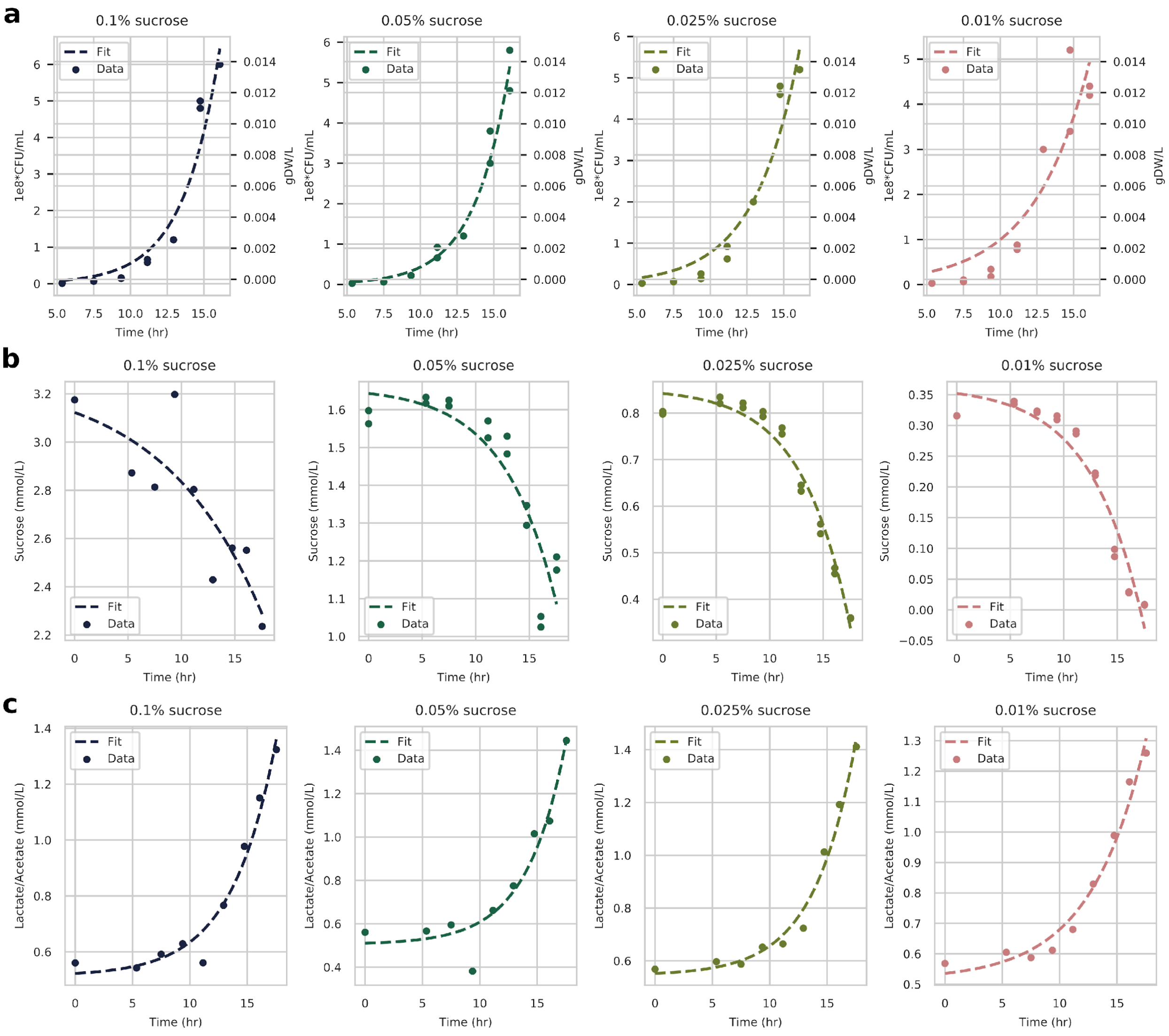

Appendix Figure S5. Raw data used to infer growth rates, sucrose uptake and lactate/acetate secretion rates. a Biomass concentration over time of *M. florum* cultures growing in CSY medium with varying initial concentration of sucrose. Biomass was measured using colony forming units (CFU/ml; left axis) and converted to grams of dry weight (gDW/L; right axis). CFU/ml quantifications were performed in technical duplicate, except for the 16-hour data point (0.1% sucrose only) and the 13-hour data point (all conditions) due to technical issues. All data points are shown. For each graph, a simple exponential growth fit is also shown (dotted line). **b and c**

Same as panel **a**, but for sucrose (**b**) and combined lactate/acetate (**c**) concentrations measured by high performance liquid chromatography. Sucrose quantifications were performed in technical duplicate (except for the 0.1% initial sucrose concentration), whereas lactate and acetate quantifications were performed in single replicates. All data points are shown. For each graph, a simple exponential growth fit is also displayed (dotted line).

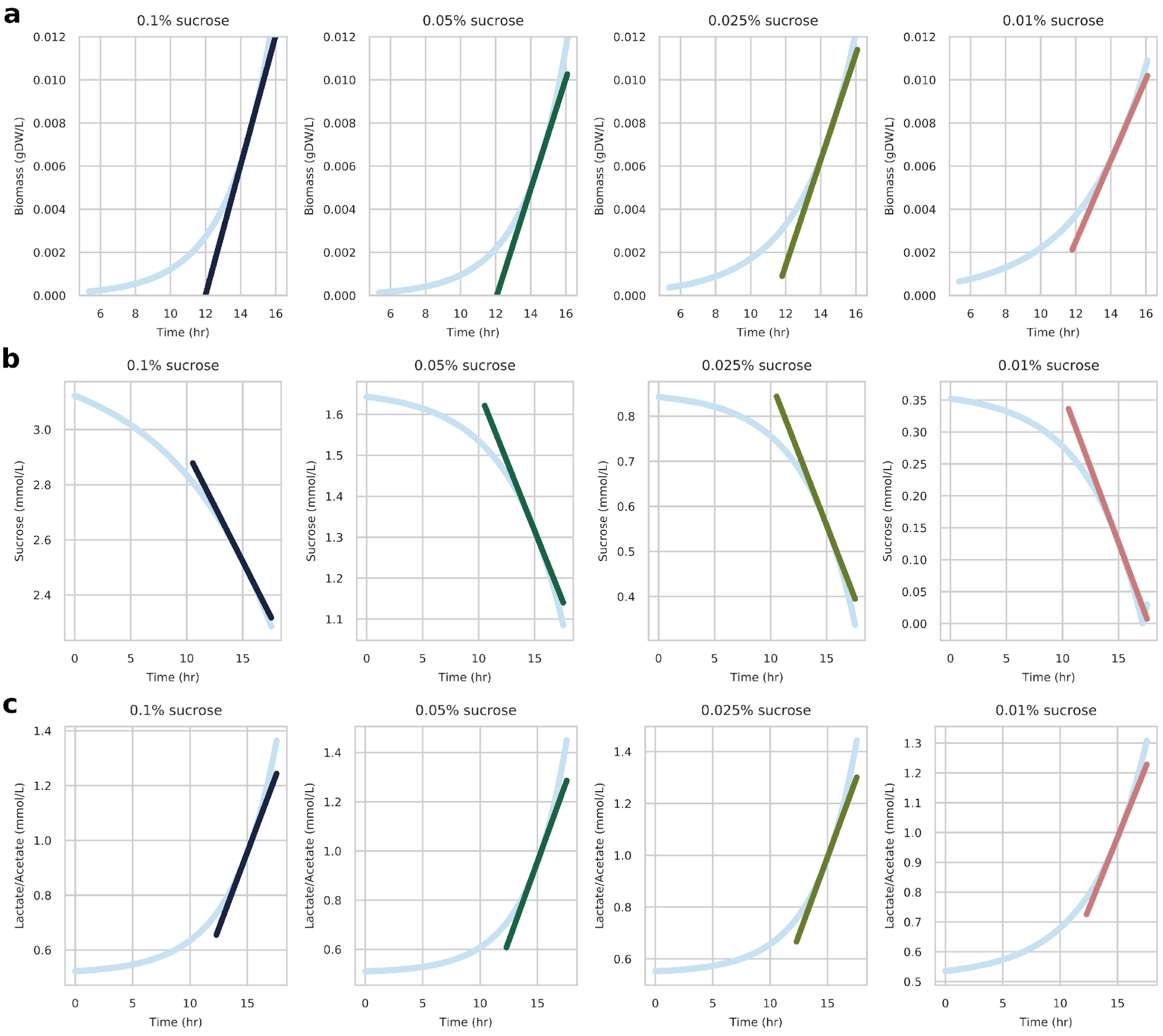

Appendix Figure S6. Linear regression in *M. florum* exponential growth phase (14 to 16 hours) for biomass (**a**), sucrose (**b**), and combined acetate/lactate (**c**) concentrations. The slopes associated with these linear regressions were used to calculate the substrate (sucrose) and product (lactate/acetate mixture) specific rates (see Materials and Methods). The linear regression plot was extrapolated to facilitate visualization.

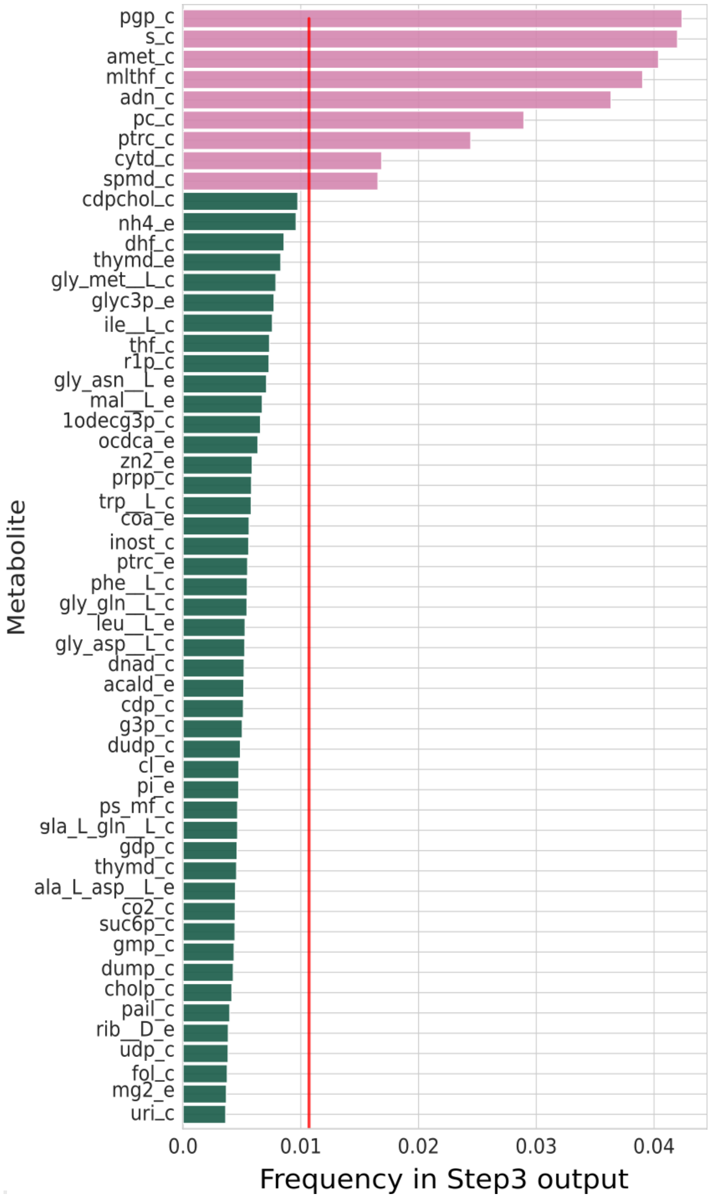

Appendix Figure S7. Metabolite apparition frequencies from the genetic algorithm output of BOFdat Step3. The nine metabolites above average (red line) shown in pink were included in the BOF (pgp, phosphatidylglycerol phosphate; s, sulfur; amet, adenosyl methionine; mlthf, 5,10-methylenetetrahydrofolate; adn, adenosine; pc, phosphatidylcholine; ptrc, putrescine; cytd, cytidine; spmd, spermidine). _c and _e suffixes indicate cytoplasm and extracellular localization, respectively. For all identifiers, please refer to the BiGG database (King *et al*, 2016).

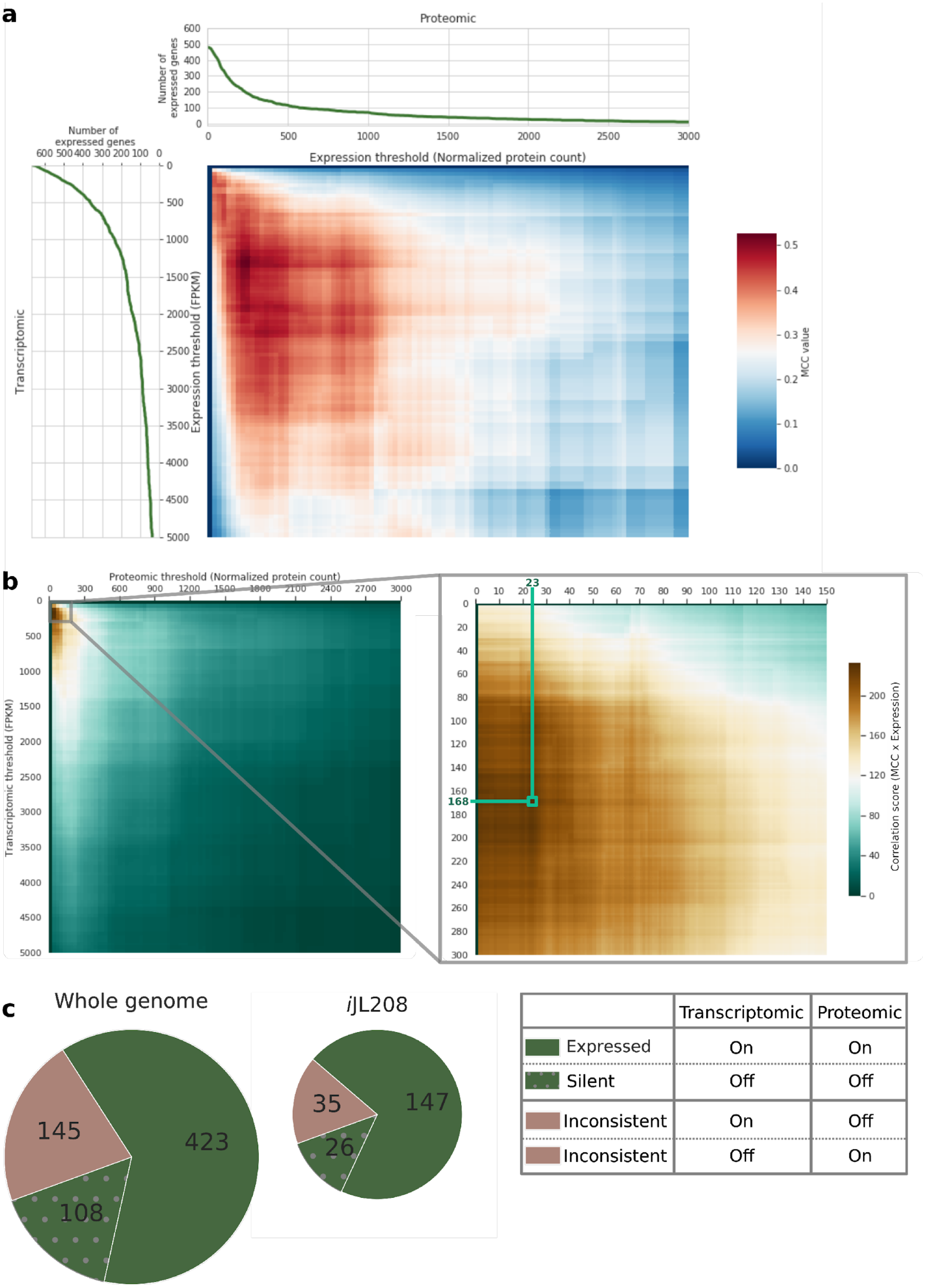
a
Proteomic
Number of expressed genes
Expression threshold (Normalized protein count)
Transcriptomic
Expression threshold (FPKM)
MCC value
b
Proteomic threshold (Normalized protein count)
Transcriptomic threshold (FPKM)
23
168
Correlation score (MCC x Expression)
c
Whole genome
145
423
108
iJL208
35
147
26
Transcriptomic
Proteomic
Expressed On On
Silent Off Off
Inconsistent On Off
Inconsistent Off On

Appendix Figure S8. Determining the optimal expression thresholds for transcriptomic and proteomic datasets. **a** Applying thresholds to recently published transcriptomic (fragments per kilobase per million of mapped reads; FPKM) and proteomic (protein molecules per cell) datasets impacts the resulting number of expressed genes (green curves). For each pair of thresholds, the Matthews correlation coefficient (MCC) comparing binary vectors of gene expression status is shown in the matrix, where high (red) and low (blue) values indicate similarity between expression status from the two experimental datasets. **b** To identify the thresholds maximizing both the expression status and the number of genes consistently expressed, the correlation values (defined by the MCC, see panel A) were multiplied by the average number of expressed genes for each threshold pair, resulting in a correlation score matrix (left). The expression thresholds selected for comparison with the predicted metabolic fluxes were 23 proteins per cell for the proteomic experiment and an FPKM of 168 for the transcriptomic experiment (right), for a total of 423 genes considered expressed in both datasets. **c** Proportion of expressed genes in the entire genome (left) and in *i*JL208 (middle) according to both proteomic and transcriptomic data and selected thresholds**.** In *i*JL208, a total of 173 genes had a consistent expression status in both datasets.

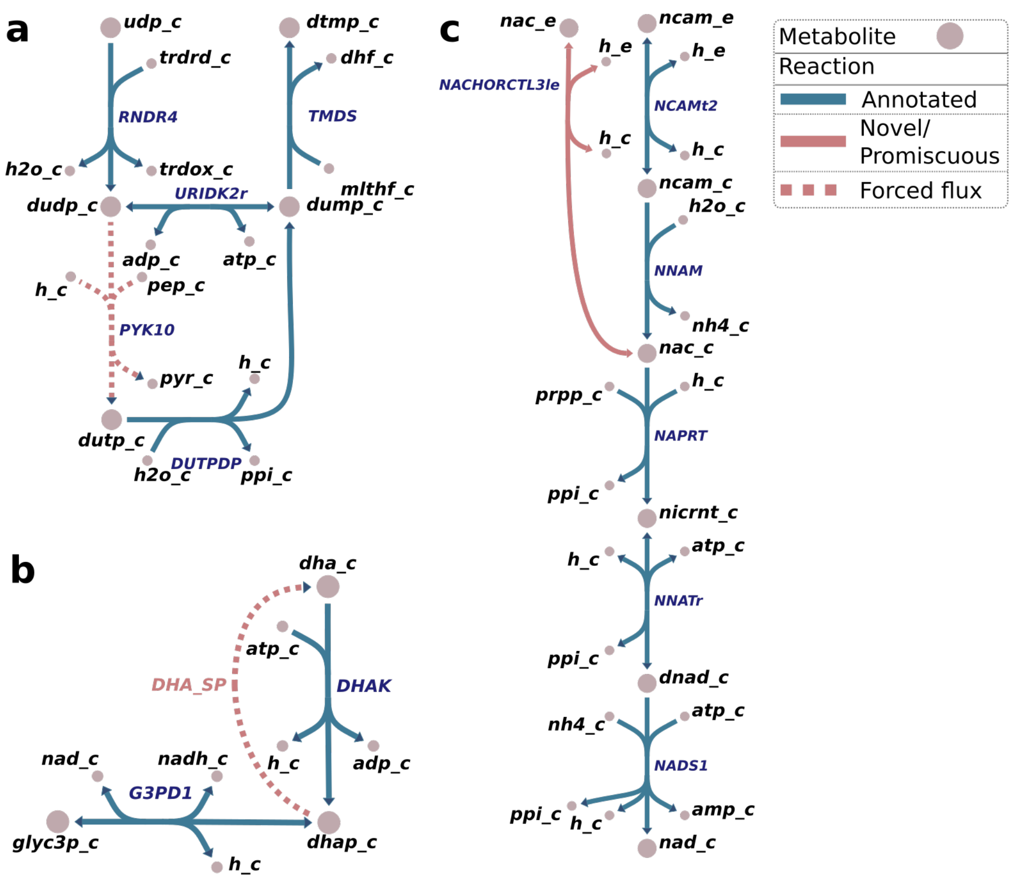

Appendix Figure S9. Resolving false negative and false positive predictions. a Forcing a flux through the PYK10 reaction simulated the production of deoxyuridine triphosphate (dutp), ensuring that the dUTPase carried flux and was essential. **b** Forcing the spontaneous production of dihydroxyacetone (dha) ensured that the DHAK carried flux and was essential. **c** Solving the only true false positive identified, the nicotinamidase Mfl340. Adding a transport reaction for nicotinate removes the need for nicotinamidase while keeping the necessity for the rest of the pathway, suggesting that nicotinate is readily available in the growth media.

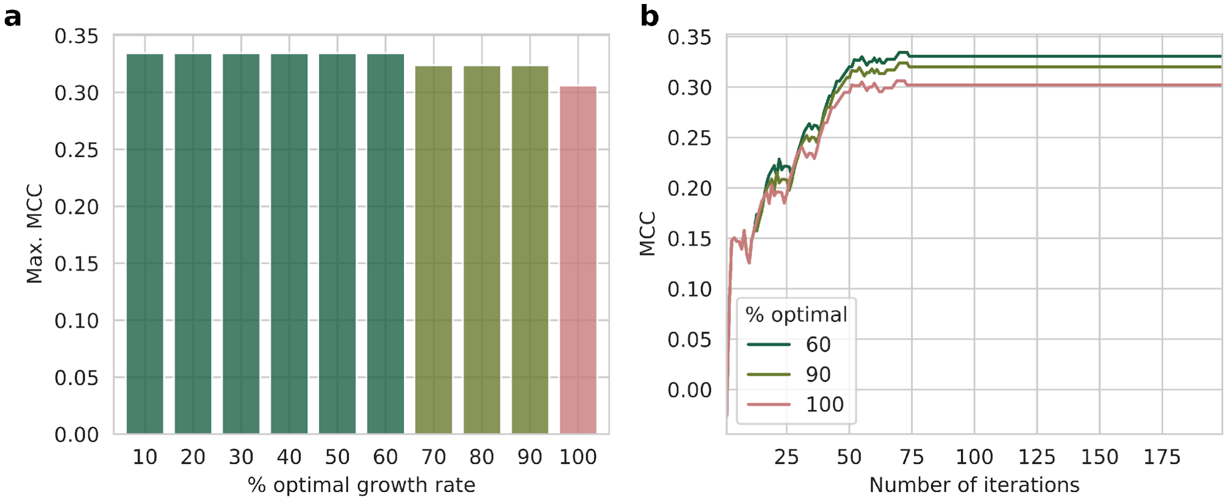

Appendix Figure S10. Impact of growth rate on reduced genome similarity with that of JCVI-syn3.0. a Maximal Matthews Correlation Coefficient (MCC) observed between JCVI-syn3.0 and different *M. florum* genome reduction possibilities generated by the MinGenome algorithm with increasing growth rate constraints. Three different scenarios were identified based on their impact on the similarity with JCVI-syn3.0 (dark green: Low, 60% growth rate; light green: Intermediate, 90% growth rate; red: Optimal, 100% growth rate). **b** MCC calculated after each MinGenome iteration which removes the largest possible stretch of genes in the genome. The three scenarios identified in **a** are presented.

Appendix Tables

Appendix Table S1. Characteristics of common Mollicutes species.

Bacterial species	Genome size (bp)*	Number of coding genes*	Natural host*	Pathogenicity*	Doubling time in rich medium
***Mesoplasma florum* L1 (AE017263)**	793,224	685	Insects or flowers	No	0.6 hr**
**JCVI Syn3.0 (CP014940.1)**	531,490	452	N/A	N/A	3 hr (Hutchison *et al*, 2016)
***Mycoplasma mycoides* JCVI-syn1.0 (CP002027.1)**	1,203,804	1,138	Cattle	Yes	1 hr (Gibson *et al*, 2010)
***Mycoplasma genitalium* G37 (AAGX00000000)**	559,388	653	Human	Yes	12 hr (Peterson & Fraser, 2001)
***Mycoplasma pneumoniae* M129 (U00089)**	816,394	755	Human	Yes	6 to 20 hr (Wodke *et al*, 2013; Peterson & Fraser, 2001)
***Mycoplasma gallisepticum* str. F (CP001873)**	978,612	796	Chicken	Yes	2 hr (Quinlan & Maniloff, 1973)
***Acholesplasma laidlawii* NCTC10116 (LS483439)**	1,498,557	1,373	N/A	No	Weeks (Windsor *et al*, 2010)

*Obtained from the PATRIC database (https://www.patricbrc.org/) (Wattam *et al*, 2017).
**This study.

Appendix Table S2. *In silico* minimal medium composition.

<table>
<tr><th>Carbon sources</th><th colspan="2">Amino acids</th><th>Vitamins</th><th>Minerals</th></tr>
<tr><td>1. D-Fructose
2. Sucrose
3. D-galactose</td><td>4. N-L-Alanyl-L-leucine
5. N-L-alanyl-L-threonine
6. L-Arginine
7. L-Asparagine
8. Cys-Gly
9. L-Glutamate
10. N-glycyl-L-aspartic acid
11. L-Glycylglutamine</td><td>12. L-Histidine
13. L-Isoleucine
14. L-Methionine
15. L-Lysine
16. L-Tryptophan
17. L-Tyrosine
18. L-Valine
19. L-Serine
20. L-Proline
21. L-Phenylalanine</td><td>22. Coenzyme A
23. Folate
24. Nicotinamide</td><td>25. Calcium
26. Co2+
27. Chloride
28. magnesium
29. Mn2+
30. Molybdate
31. Sodium
32. potassium
33. Nickel
34. Zinc</td></tr>
<tr><th>Nucleotides</th><th colspan="2">Lipids</th><th colspan="2">Polyamines</th></tr>
<tr><td>35. Adenine
36. Guanine
37. Uracil
38. Thymidine</td><td colspan="2">39. Octadecanoate (n-C18:0)
40. Glycerol 3-phosphate
41. Choline
42. N-acylsphingosine/Ceramide</td><td colspan="2">43. Spermidine
44. Putrescine</td></tr>
</table>

Appendix Table S3. Final *i*JL208 biomass composition.

<table>
<tr><th colspan="3">Proteins (46.6%)</th><th colspan="2">Others (1.2%)</th></tr>
<tr><td colspan="2">1. L-Alanyl-tRNA(Ala)
2. L-Arginyl-tRNA(Arg)
3. L-Asparaginyl-tRNA(Asn)
4. L-Aspartyl-tRNA(Asp)
5. L-Cysteinyl-tRNA(Cys)
6. L-Glutaminyl-tRNA(Gln)
7. L-Glutamyl-tRNA(Glu)
8. L-Valyl-tRNA(Val)
9. Glycyl-tRNA(Gly)</td><td>10. L-Histidyl-RNA(His)
11. L-Isoleucyl-tRNA(Ile)
12. L-Leucyl-tRNA(Leu)
13. L-Lysine-tRNA (Lys)
14. L-Methionyl-tRNA (Met)
15. L-Phenylalanyl-tRNA(Phe)
16. L-Prolyl-tRNA(Pro)
17. L-Seryl-tRNA(Ser)
18. L-Threonyl-tRNA(Thr)
19. L-Tryptophanyl-tRNA(Trp)
20. L-Tyrosyl-tRNA(Tyr)</td><td>21. S-Adenosyl-L-methionine
22. 5,10-Methylenetetrahydrofolate
23. Nicotinamide adenine dinucleotide
24. Nicotinamide adenine dinucleotide - reduced
25. Nicotinamide adenine dinucleotide phosphate
26. Nicotinamide adenine dinucleotide phosphate – reduced
27. Phosphate
28. Manganese
29. Molybdate
30. Sodium
31. Magnesium</td><td>32. Ammonium
33. Nickel
34. Putrescine
35. Spermidine
36. Sulfur
37. Adenosine
38. Cytidine
39. Zinc
40. Calcium
41. Choline phosphate
42. Chloride
43. Potassium
44. Co2+
45. H2O
46. Acyl carrier protein
47. Adenosine diphosphate</td></tr>
<tr><th>RNA (22.9%)</th><th>DNA (7.7%)</th><th>Lipids (18.3%)</th><th colspan="2">Glycans (4.1%)</th></tr>
<tr><td>48. ATP
49. CTP
50. UTP
51. GTP</td><td>52. dATP
53. dCTP
54. dGTP
55. dTTP</td><td>56. Phosphatidylcholine
57. Phosphatidylglycerophosphate</td><td colspan="2">58. Capsular polysaccharide Mesoplasma florum</td></tr>
</table>

Appendix Table S4. Main candidates following structural comparison by FATCAT 2.0.

Locus tag	Original RefSeq annotation	PDB match	FATCAT p-value	Suggested promiscuous reaction catalyzed
**Mfl499**	Trehalose-6-phosphate hydrolase (TRE6PH)	5zcbA	0.00*	Hydrolysis of maltose-6-phosphate to produce glucose and glucose-6-phosphate
**Mfl254**	Glucose-6-phosphate isomerase (PGI)	1tzbA	8.68e-12	Conversion of mannose-6-phosphate to fructose-6-phosphate
**Mfl120**	Phosphomannomutase (PMANM)	1k2yX	0.00*	Conversion of glucose-6-phosphate to glucose-1-phosphate
**Mfl121**	2-deoxyribose-5-phosphate aldolase (DRPA)	1vpx	5.96e-10	Pentose phosphate pathway transaldolase
**Mfl639**	2-deoxyribose-5-phosphate aldolase (DRPA)	1vpx	2.51e-9	Pentose phosphate pathway transaldolase

*A p-value of 0.00 means that the PDB match was used as a template by I-TASSER for reconstruction of the 3D structure.

Appendix References

Acevedo-Rocha CG, Fang G, Schmidt M, Ussery DW & Danchin A (2013) From essential to persistent genes: a functional approach to constructing synthetic life. *Trends Genet* 29: 273–279

AlQuraishi M (2019) AlphaFold at CASP13. *Bioinformatics* 35: 4862–4865

Antczak M, Michaelis M & Wass MN (2019) Environmental conditions shape the nature of a minimal bacterial genome. *Nat Commun* 10: 3100

Artimo P, Jonnalagedda M, Arnold K, Baratin D, Csardi G, de Castro E, Duvaud S, Flegel V, Fortier A, Gasteiger E, *et al* (2012) ExPASy: SIB bioinformatics resource portal. *Nucleic Acids Res* 40: W597-603

Auiewiriyanukul W, Saburi W, Kato K, Yao M & Mori H (2018) Function and structure of GH13_31 α-glucosidase with high α-(1→4)-glucosidic linkage specificity and transglucosylation activity. *FEBS Letters* 592: 2268–2281

Baby V, Lachance J-C, Gagnon J, Lucier J-F, Matteau D, Knight T & Rodrigue S (2018) Inferring the Minimal Genome of *Mesoplasma florum* by Comparative Genomics and Transposon Mutagenesis. *mSystems* 3: e00198-17

Barré A, de Daruvar A & Blanchard A (2004) MolliGen, a database dedicated to the comparative genomics of Mollicutes. *Nucleic Acids Res* 32: D307-10

Bautista EJ, Zinski J, Szczepanek SM, Johnson EL, Tulman ER, Ching W-M, Geary SJ & Srivastava R (2013) Semi-automated Curation of Metabolic Models via Flux Balance Analysis: A Case Study with *Mycoplasma gallisepticum*. *PLoS Computat* 9: e1003208

Ben-Menachem G, Himmelreich R, Herrmann R, Aharonowitz Y & Rottem S (1997) The thioredoxin reductase system of mycoplasmas. *Microbiology* 143 (Pt 6): 1933–1940

Berman HM, Westbrook J, Feng Z, Gilliland G, Bhat TN, Weissig H, Shindyalov IN & Bourne PE (2000) The Protein Data Bank. *Nucleic Acids Res* 28: 235–242

Bertin C, Pau-Roblot C, Courtois J, Manso-Silván L, Tardy F, Poumarat F, Citti C, Sirand-Pugnet P, Gaurivaud P & Thiaucourt F (2015) Highly dynamic genomic loci drive the synthesis of two types of capsular or secreted polysaccharides within the *Mycoplasma mycoides* cluster. *Appl Environ Microbiol* 81: 676–687

Bertin C, Pau-Roblot C, Courtois J, Manso-Silván L, Thiaucourt F, Tardy F, Le Grand D, Poumarat F & Gaurivaud P (2013) Characterization of free exopolysaccharides secreted by *Mycoplasma mycoides* subsp. *mycoides*. *PLoS One* 8: e68373

Béven L, Charenton C, Dautant A, Bouyssou G, Labroussaa F, Sköllermo A, Persson A, Blanchard A & Sirand-Pugnet P (2012) Specific evolution of F1-like ATPases in mycoplasmas. *PLoS One* 7: e38793

Billings WM, Hedelius B, Millecam T, Wingate D & Della Corte D (2019) ProSPr: Democratized Implementation of Alphafold Protein Distance Prediction Network. *bioRxiv*

Bizarro CV & Schuck DC (2007) Purine and pyrimidine nucleotide metabolism in Mollicutes. *Genet Mol Biol* 30: 190–201

Breuer M, Earnest TM, Merryman C, Wise KS, Sun L, Lynott MR, Hutchison CA, Smith HO, Lapek JD, Gonzalez DJ, *et al* (2019) Essential metabolism for a minimal cell. *Elife* 8: 1–77

Browning G & Citti C (2014) Mollicutes: Molecular Biology and Pathogenesis Poole, England: Caister Academic Press

Byers DM & Gong H (2007) Acyl carrier protein: structure-function relationships in a conserved multifunctional protein family. *Biochem Cell Biol* 85: 649–662

Danchin A & Fang G (2016) Unknown unknowns: essential genes in quest for function. *Microb Biotechnol* 9: 530–540

Ebrahim A, Lerman JA, Palsson BO & Hyduke DR (2013) COBRApy: COnstraints-Based Reconstruction and Analysis for Python. *BMC Syst Biol* 7: 74

Fraser CM, Gocayne JD, White O, Adams MD, Clayton RA, Fleischmann RD, Bult CJ, Kerlavage AR, Sutton G, Kelley JM, *et al* (1995) The minimal gene complement of *Mycoplasma genitalium*. *Science* 270: 397–403

Ghatak S, King ZA, Sastry A & Palsson BO (2019) The y-ome defines the 35% of *Escherichia coli* genes that lack experimental evidence of function. *Nucleic Acids Res* 47: 2446–2454

Gibson DG, Glass JI, Lartigue C, Noskov VN, Chuang R-Y, Algire MA, Benders GA, Montague MG, Ma L, Moodie MM, *et al* (2010) Creation of a bacterial cell controlled by a chemically synthesized genome. *Science* 329: 52–56

Glass JI, Assad-Garcia N, Alperovich N, Yooseph S, Lewis MR, Maruf M, Hutchison CA 3rd, Smith HO & Venter JC (2006) Essential genes of a minimal bacterium. *Proc Natl Acad Sci U S A* 103: 425–430

Glass JI, Merryman C, Wise KS, Hutchison CA & Smith HO (2017) Minimal Cells—Real and Imagined. *Cold Spring Harb Perspect Biol* 1: 1–12

Hackett KJ, Ginsberg AS, Rottem S, Henegar RB & Whitcomb RF (1987) A defined medium for a fastidious *Spiroplasma*. *Science* 237: 525–527

Heath RJ, Jackowski S & Rock CO (2002) Fatty acid and phospholipid metabolism in prokaryotes. In *Biochemistry of Lipids, Lipoproteins and Membranes*, Vance DE & Vance JE (eds) pp 55–92. ELSEVIER

Hirokawa Y, Kawano H, Tanaka-Masuda K, Nakamura N, Nakagawa A, Ito M, Mori H, Oshima T & Ogasawara N (2013) Genetic manipulations restored the growth fitness of reduced-genome *Escherichia coli*. *J Biosci Bioeng* 116: 52–58

Hong Y & Reeves PR (2014) Diversity of o-antigen repeat unit structures can account for the substantial sequence variation of wzx translocases. *J Bacteriol* 196: 1713–1722

Hosie AH & Poole PS (2001) Bacterial ABC transporters of amino acids. *Res Microbiol* 152: 259–270

Hutchison CA 3rd, Chuang R-Y, Noskov VN, Assad-Garcia N, Deerinck TJ, Ellisman MH, Gill J, Kannan K, Karas BJ, Ma L, *et al* (2016) Design and synthesis of a minimal bacterial genome. *Science* 351: aad6253

Kanehisa M, Furumichi M, Tanabe M, Sato Y & Morishima K (2017) KEGG: new perspectives on genomes, pathways, diseases and drugs. *Nucleic Acids Res* 45: D353–D361

Kanehisa M & Goto S (2000) KEGG: kyoto encyclopedia of genes and genomes. *Nucleic Acids Res* 28: 27–30

Kanehisa M, Sato Y, Kawashima M, Furumichi M & Tanabe M (2016) KEGG as a reference resource for gene and protein annotation. *Nucleic Acids Res* 44: D457-62

King ZA, Lu J, Dräger A, Miller P, Federowicz S, Lerman JA, Ebrahim A, Palsson BO & Lewis NE (2016) BiGG Models: A platform for integrating, standardizing and sharing genome-scale models. *Nucleic Acids Res* 44: D515-22

Kumar VS & Maranas CD (2009) GrowMatch: an automated method for reconciling *in silico/in vivo* growth predictions. *PLoS Comput Biol* 5: e1000308

Lachance JC, Lloyd CJ, Monk JM, Yang L, Sastry AV, Seif Y, Palsson BO, Rodrigue S, Feist AM, King ZA, *et al* (2019) BOFdat: Generating biomass objective functions for genome-scale metabolic models from experimental data. *PLoS Comput Biol* 15: e1006971

Lazarev VN, Levitskii SA, Basovskii YI, Chukin MM, Akopian TA, Vereshchagin VV, Kostrjukova ES, Kovaleva GY, Kazanov MD, Malko DB, *et al* (2011) Complete genome and proteome of *Acholeplasma laidlawii*. *J Bacteriol* 193: 4943–4953

Lewis NE, Hixson KK, Conrad TM, Lerman JA, Charusanti P, Polpitiya AD, Adkins JN, Schramm G, Purvine SO, Lopez-Ferrer D, *et al* (2010) Omic data from evolved *E. coli* are consistent with computed optimal growth from genome-scale models. *Mol Syst Biol* 6: 390

Li Z, Jaroszewski L, Iyer M, Sedova M & Godzik A (2020) FATCAT 2.0: towards a better understanding of the structural diversity of proteins. *Nucleic Acids Research* 48: W60–W64

Lu F, Li S, Jiang Y, Jiang J, Fan H, Lu G, Deng D, Dang S, Zhang X, Wang J, *et al* (2011) Structure and mechanism of the uracil transporter UraA. *Nature* 472: 243–246

Machado D & Herrgård M (2014) Systematic evaluation of methods for integration of transcriptomic data into constraint-based models of metabolism. *PLoS Comput Biol* 10: e1003580

Matteau D, Lachance J-C, Grenier F, Gauthier S, Daubenspeck JM, Dybvig K, Garneau D, Knight TF, Jacques P-É & Rodrigue S (2020) Integrative characterization of the near-minimal bacterium *Mesoplasma florum*. *Mol Syst Biol* 16: e9844

McCoy RE, Basham HG, Tully JG, Rose DL, Carle P & Bové JM (1984) *Acholeplasma florum*, a New Species Isolated from Plants. *Int J Syst Bacteriol* 34: 11–15

Mih N, Brunk E, Chen K, Catoiu E, Sastry A, Kavvas E, Monk JM, Zhang Z & Palsson BO (2018) ssbio: a Python framework for structural systems biology. *Bioinformatics* 34: 2155–2157

Miles RJ (1992) Catabolism in mollicutes. *J Gen Microbiol* 138: 1773–1783

Mohamed ET, Mundhada H, Landberg J, Cann I, Mackie RI, Nielsen AT, Herrgård MJ & Feist AM (2019) Generation of an *E. coli* platform strain for improved sucrose utilization using adaptive laboratory evolution. *Microb Cell Fact* 18: 116

Moretti S, Martin O, Van Du Tran T, Bridge A, Morgat A & Pagni M (2016) MetaNetX/MNXref--reconciliation of metabolites and biochemical reactions to bring together genome-scale metabolic networks. *Nucleic Acids Res* 44: D523-6

Morowitz HJ (1984) The completeness of molecular biology. *Israel journal of medical* 20: 750–753

Neyrolles O, Brenner C, Prevost MC, Fontaine T, Montagnier L & Blanchard A (1998) Identification of two glycosylated components of *Mycoplasma penetrans*: a surface-exposed capsular polysaccharide and a glycolipid fraction. *Microbiology* 144 (Pt 5): 1247–1255

Norsigian CJ, Pusarla N, McConn JL, Yurkovich JT, Dräger A, Palsson BO & King Z (2020) BiGG Models 2020: multi-strain genome-scale models and expansion across the phylogenetic tree. *Nucleic Acids Res* 48: D402–D406

Oshida K, Shimizu T, Takase M, Tamura Y, Shimizu T & Yamashiro Y (2003) Effects of Dietary Sphingomyelin on Central Nervous System Myelination in Developing Rats. *Pediatric Research* 53: 589–593

Peterson SN & Fraser CM (2001) The complexity of simplicity. *Genome Biol* 2: COMMENT2002.1

Placzek S, Schomburg I, Chang A, Jeske L, Ulbrich M, Tillack J & Schomburg D (2017) BRENDA in 2017: new perspectives and new tools in BRENDA. *Nucleic Acids Res* 45: D380–D388

Pollack JD (2002) The necessity of combining genomic and enzymatic data to infer metabolic function and pathways in the smallest bacteria: amino acid, purine and pyrimidine metabolism in Mollicutes. *Frontiers in Bioscience* 7: d1762-1781

Pollack JD, Myers MA, Dandekar T & Herrmann R (2002) Suspected Utility of Enzymes with Multiple Activities in the Small Genome *Mycoplasma* Species: The Replacement of the Missing " Household" Nucleoside Diphosphate Kinase Gene and Activity by Glycolytic Kinases. *OMICS: A Journal of Integrative Biology* 6: 247–258

Pollack JD, Tryon VV & Beaman KD (1983) The metabolic pathways of *Acholeplasma* and *Mycoplasma*: an overview. *Yale J Biol Med* 56: 709–716

Pollack JD & Williams MV (1996) Comparative Metabolism of *Mesoplasma, Entomoplasma, Mycoplasma*, and *Acholeplasma*. *Int J*: 885–890

Pollack JD, Williams MV & McElhaney RN (1997) The comparative metabolism of the mollicutes (Mycoplasmas): the utility for taxonomic classification and the relationship of putative gene annotation and phylogeny to enzymatic function in the smallest free-living cells. *Crit Rev Microbiol* 23: 269–354

Quinlan DC & Maniloff J (1973) Deoxyribonucleic acid synthesis in synchronously growing *Mycoplasma gallisepticum*. *J Bacteriol* 115: 117–120

Regni C, Tipton PA & Beamer LJ (2002) Crystal structure of PMM/PGM: an enzyme in the biosynthetic pathway of *P. aeruginosa* virulence factors. *Structure* 10: 269–279

Reuß DR, Altenbuchner J, Mäder U, Rath H, Ischebeck T, Sappa PK, Thürmer A, Guérin C, Nicolas P, Steil L, *et al* (2017) Large-scale reduction of the *Bacillus subtilis* genome: consequences for the transcriptional network, resource allocation, and metabolism. *Genome Res* 27: 289–299

Roy A, Kucukural A & Zhang Y (2010) I-TASSER: a unified platform for automated protein structure and function prediction. *Nat Protoc* 5: 725–738

Saito Y, Silvius JR & McElhaney RN (1978) Membrane lipid biosynthesis in *Acholeplasma laidlawii* b: elongation of medium- and long-chain exogenous fatty acids in growing cells. *J Bacteriol* 133: 66–74

Salaemae W, Booker GW & Polyak SW (2016) The Role of Biotin in Bacterial Physiology and Virulence: a Novel Antibiotic Target for *Mycobacterium tuberculosis*. *Microbiol Spectr* 4

Salman M & Rottem S (1995) The cell membrane of *Mycoplasma penetrans*: lipid composition and phospholipase A1 activity. *Biochim Biophys Acta* 1235: 369–377

Schilling CH, Thakar R, Travnik E, Van Dien S & Wiback S SimPheny^{TM}: A Computational Infrastructure for Systems Biology. *CiteSeerX*

Schlame M (2008) Cardiolipin synthesis for the assembly of bacterial and mitochondrial membranes. *J Lipid Res* 49: 1607–1620

Seelig B (2017) Multifunctional enzymes from reduced genomes - model proteins for simple primordial metabolism? *Mol Microbiol* 105: 505–507

Senior AW, Evans R, Jumper J, Kirkpatrick J, Sifre L, Green T, Qin C, Žídek A, Nelson AWR, Bridgland A, *et al* (2019) Protein structure prediction using multiple deep neural networks in CASP13. *Proteins: Struct Funct Bioinf* 87: 1141–1148

Suthers PF, Dasika MS, Kumar VS, Denisov G, Glass JI & Maranas CD (2009) A Genome-Scale Metabolic Reconstruction of *Mycoplasma genitalium*, iPS189. *PLoS Comput Biol* 5: e1000285

Swan MK, Hansen T, Schönheit P & Davies C (2004) A Novel Phosphoglucose Isomerase (PGI)/Phosphomannose Isomerase from the *Crenarchaeon Pyrobaculum aerophilum* Is a Member of the PGI Superfamily: structural evidence at 1.16-A resolution. *J Biol Chem* 279: 39838–39845

Thiele I & Palsson BØ (2010) A protocol for generating a high-quality genome-scale metabolic reconstruction. *Nat Protoc* 5: 93–121

Vinitsky A & Grubmeyer C (1993) A new paradigm for biochemical energy coupling. *Salmonella typhimurium* nicotinate phosphoribosyltransferase. *J Biol Chem* 268: 26004–26010

Wang L & Maranas CD (2018) MinGenome: An In Silico Top-Down Approach for the Synthesis of Minimized Genomes. *ACS Synth Biol* 7: 462–473

Wattam AR, Davis JJ, Assaf R, Boisvert S, Brettin T, Bun C, Conrad N, Dietrich EM, Disz T, Gabbard JL, *et al* (2017) Improvements to PATRIC, the all-bacterial Bioinformatics Database and Analysis Resource Center. *Nucleic Acids Res* 45: D535–D542

Weiner J 3rd, Zimmerman C-U, Göhlmann HWH & Herrmann R (2003) Transcription profiles of

the bacterium *Mycoplasma pneumoniae* grown at different temperatures. *Nucleic Acids Res* 31: 6306–6320

Westphal LL, Sauvey P, Champion MM, Ehrenreich IM & Finkel SE (2016) Genomewide Dam Methylation in *Escherichia coli* during Long-Term Stationary Phase. *mSystems* 1: e00130-16

Windsor HM, Windsor GD & Noordergraaf JH (2010) The growth and long term survival of *Acholeplasma laidlawii* in media products used in biopharmaceutical manufacturing. *Biologicals* 38: 204–210

Wodke JAH, Puchałka J, Lluch-Senar M, Marcos J, Yus E, Godinho M, Gutiérrez-Gallego R, dos Santos VAPM, Serrano L, Klipp E, *et al* (2013) Dissecting the energy metabolism in *Mycoplasma pneumoniae* through genome-scale metabolic modeling. *Mol Syst Biol* 9: 653

Xavier JC, Patil KR & Rocha I (2017) Integration of Biomass Formulations of Genome-Scale Metabolic Models with Experimental Data Reveals Universally Essential Cofactors in Prokaryotes. *Metab Eng* 39: 200–208

Yang J, Yan R, Roy A, Xu D, Poisson J & Zhang Y (2015) The I-TASSER Suite: protein structure and function prediction. *Nat Methods* 12: 7–8

Yang Z & Tsui SK-W (2018) Functional Annotation of Proteins Encoded by the Minimal Bacterial Genome Based on Secondary Structure Element Alignment. *J Proteome Res* 17: 2511–2520

Yang Z, Zeng X & Tsui SK-W (2019) Investigating function roles of hypothetical proteins encoded by the *Mycobacterium tuberculosis* H37Rv genome. *BMC Genomics* 20: 394

Yus E, Maier T, Michalodimitrakis K, van Noort V, Yamada T, Chen W-H, Wodke JAH, Güell M, Martínez S, Bourgeois R, *et al* (2009) Impact of genome reduction on bacterial metabolism and its regulation. *Science* 326: 1263–1268

Zhang C, Freddolino PL & Zhang Y (2017) COFACTOR: improved protein function prediction by combining structure, sequence and protein–protein interaction information. *Nucleic Acids Res* 45: W291–W299
