# Supplementary material for: Genome‐scale metabolic modeling reveals key features of a minimal gene set
Source: Mol Syst Biol. 2021 Jul 20;17(7):e10099. doi: 10.15252/msb.202010099 (PMC8290834; doi:10.15252/msb.202010099)
Supplement: Supplementary file 2 — Expanded View Figures PDF [file MSB-17-e10099-s011.pdf]

Expanded View Figures

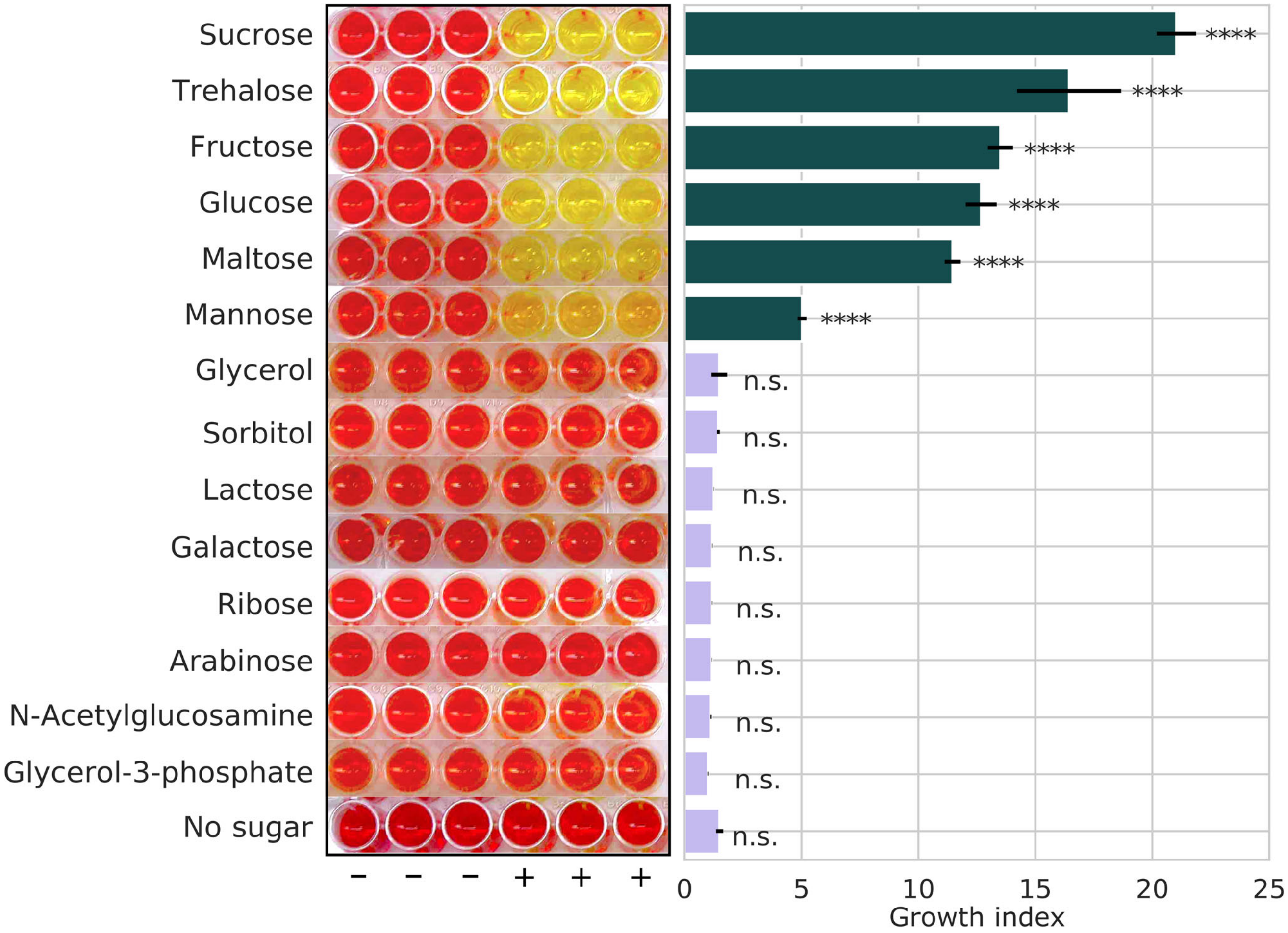

Figure EV1. Experimental evaluation of *Mesoplasma florum* growth on different carbohydrates.

The phenol red, a pH indicator present in the CSY medium, changes color upon metabolic activity. The medium color ($OD_{560\ nm}$) observed after a 24-h incubation period and normalized over a non-inoculated control, corresponding to the growth index, is reported for each carbohydrate tested in CSY. Carbohydrates were supplemented at 1% (w/v) final concentration. Bars and error bars indicate the mean and standard deviation calculated from technical triplicate, respectively (one-way ANOVA; ****$P < 0.0001$).

© 2021 The Authors

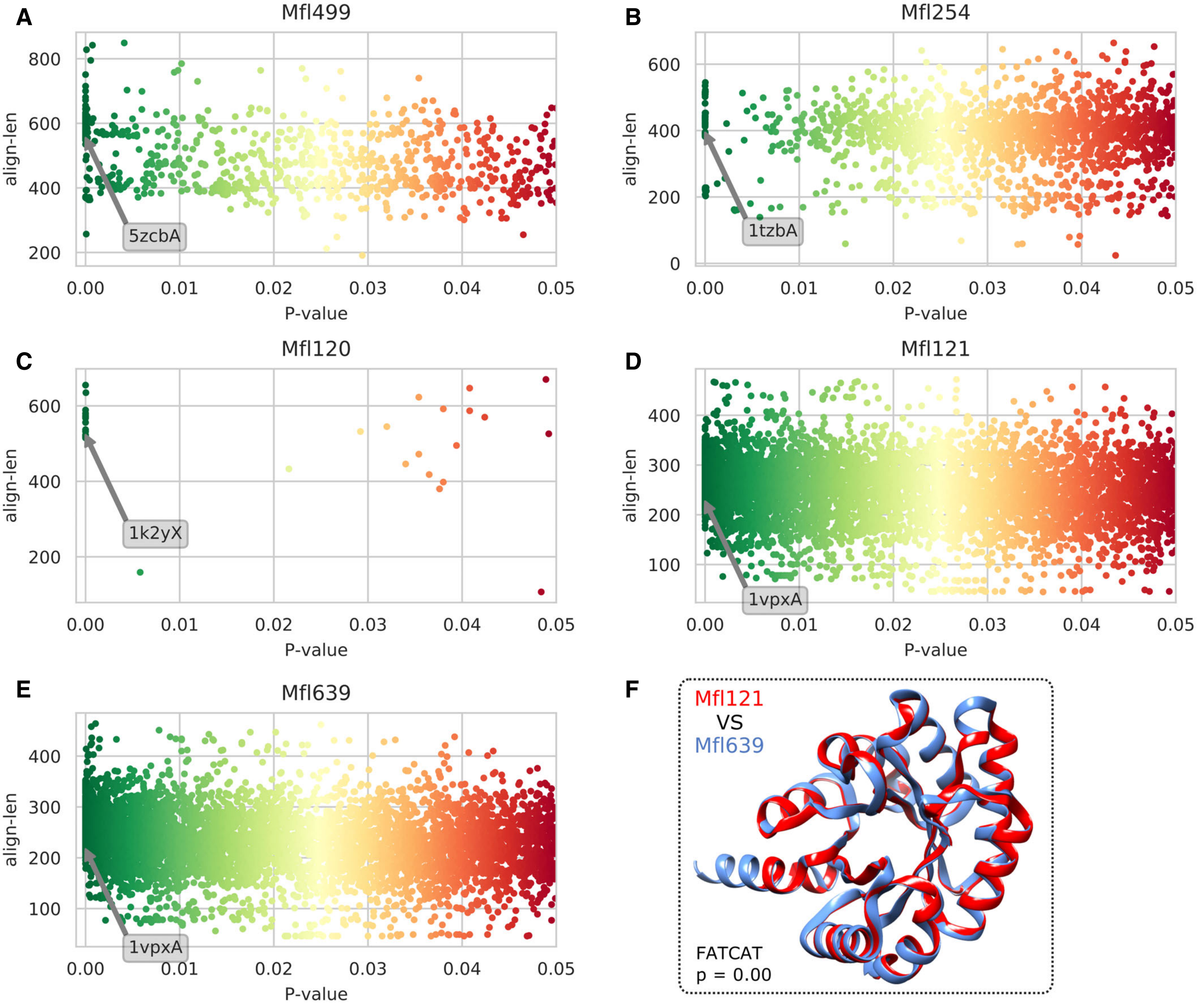

Figure EV2. FATCAT 2.0 database alignment results.

On each plot, significant alignments ($P < 0.05$) between a selected *Mesoplasma florum* candidate protein and the Protein Data Bank are shown. Alignments are separated according to their respective length (align-len) and associated *P*-value (colored from red to green) generated by the FATCAT 2.0 server (Li *et al*, 2020).

A Alignment results for the Mfl499 protein. The arrow indicates a positive match with the A chain of the α-glucosidase of *Bacillus* sp. AHU2216 (5zcbA; *Bsp*AG13_31A) specific to α-(1-4)-glucosidic linkage.

B–E Same as panel (A) but for (B) the Mfl254 protein and the A chain of the phosphoglucose/phosphomannose isomerase of *Pyrobaculum aerophilum* (1tzbA; PaPGI/PMI), (C) Mfl120 protein and the X chain of the phosphomannomutase/phosphoglucomutase of *Pseudomonas aeruginosa* (1k2yX; PMM/PGM), and (D, E) Mfl121 and Mfl639 proteins and the A chain of the transaldolase of *Thermotoga maritima* (1vpxA; TM0295), respectively.

F FATCAT alignment of the reconstructed structures of both transaldolase Mfl121 (red) and Mfl639 (blue).

Source data are available online for this figure.

 © 2021 The Authors

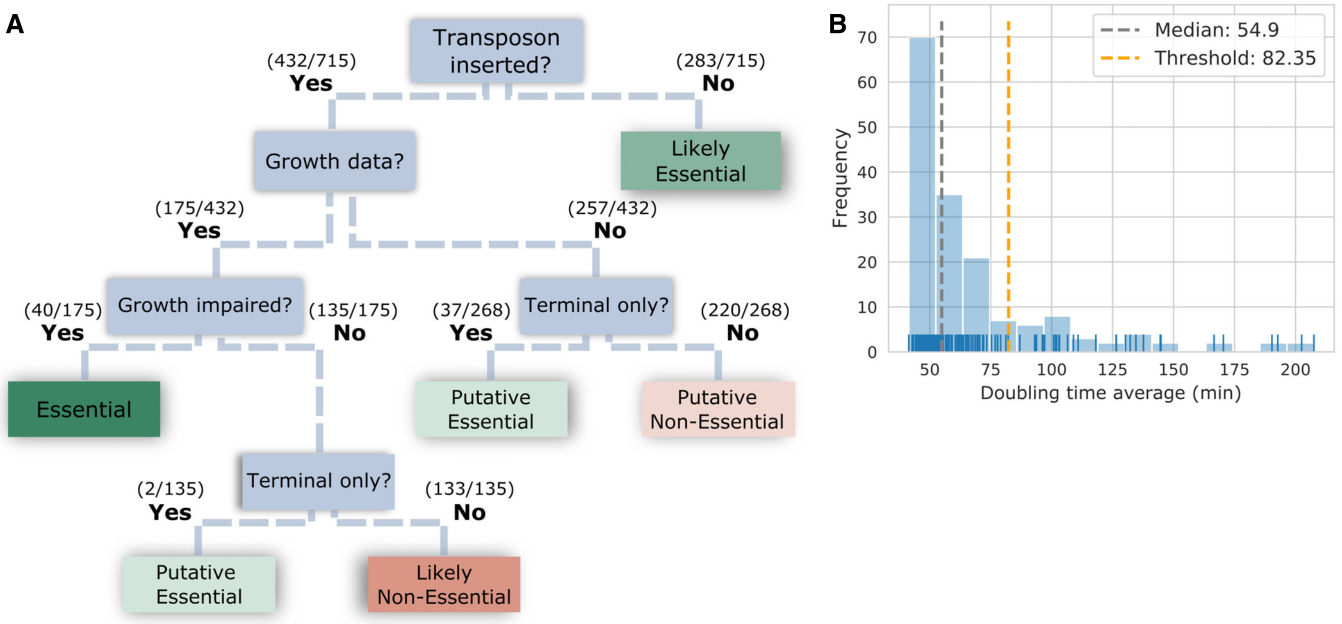

Figure EV3. Revisiting the *Mesoplasma florum* genome-wide essentiality data.

A Transposon mutagenesis experiments were previously published for *M. florum* L1, but considered only the presence or absence of a transposon insertion into a gene to determine its essentiality (Baby *et al*, 2018b). These data were re-analyzed using the presented decision tree and now accounts for the relative position of the insertion site within the interrupted gene as well as growth data of the isolated mutants. For insertions not impairing the growth of *M. florum*, interrupted genes were considered essential only if the transposons were strictly restricted to the terminal region of genes, defined as the last 20% of the gene length. In downstream analyses, all likely and putative essentials were considered as essential genes, whereas likely and putative non-essentials were considered as non-essentials.

B Defining growth impairment threshold. Histogram (blue bars) showing the distribution of doubling time for the 175 insertion mutants (blue ticks) with reliable growth data (Dataset EV5). Mutants showing a doubling time higher than the sum of the median and the median absolute deviation were considered non-viable.

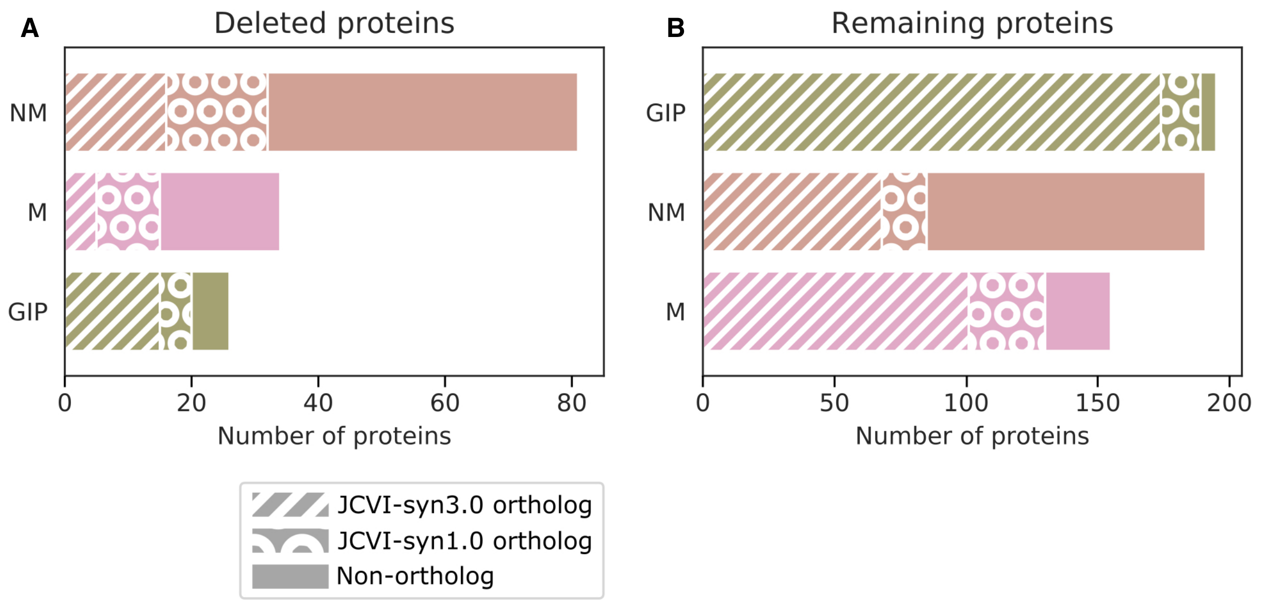

Figure EV4.

◀

Figure EV4. Distribution of most represented general KEGG categories.

A, B Deleted (A) and retained (B) proteins from the minimal genome prediction in the three most represented KEGG functional categories presented in Fig 7C. The proteins homologous to JCVI-syn3.0 are represented by white hatched bars, while the genes that were absent from JCVI-syn3.0 but homologous to JCVI-syn1.0 are represented by white circles. The plain color represents the proteins present only in *Mesoplasma florum*. NM, not mapped; GIP, genetic information processing; M, metabolism.

 © 2021 The Authors
